# MicroRNA Deregulation and Immune Checkpoint Interactions in Common Variable Immunodeficiency and CLL-Associated Secondary Immunodeficiency

**DOI:** 10.3390/cells14201577

**Published:** 2025-10-10

**Authors:** Paulina Mertowska, Sebastian Mertowski, Milena Czosnek, Barbara Sosnowska-Pasiarska, Aleksandra Krasińska-Płachta, Zbigniew Krasiński, Tomasz Urbanowicz, Krzysztof Bojarski, Mansur Rahnama-Hezavah, Ewelina Grywalska

**Affiliations:** 1 Department of Experimental Immunology, Medical University of Lublin, 20-093 Lublin, Poland; sebastian.mertowski@umlub.pl (S.M.);; 2 Student Research Group of Experimental Immunology, Medical University of Lublin, 20-093 Lublin, Poland; 3 Department of Oncocardiology, Holy Cross Cancer Center, 25-734 Kielce, Poland; 4 Department of Ophthalmology, Poznan University of Medical Sciences, Fredry 10, 61-107 Poznan, Poland; 5 Department of Vascular, Endovascular Surgery, Angiology and Phlebology, Poznan University of Medical Science, Dluga 1/2, 61-848 Poznań, Poland; 6 Department of Cardiac Surgery and Transplantology, Poznan University of Medical Sciences, 61-848 Poznań, Poland; 7 General Surgery Department, SP ZOZ in Leczna, 52 Krasnystawska Street, 21-010 Leczna, Poland; 8 Department of Dental Surgery, Medical University of Lublin, 6 Chodźki Street, 20-093 Lublin, Poland

**Keywords:** immunodeficiencies, CVID, CLL, microRNA, dPCR, immune checkpoints, biomarkers

## Abstract

**Background:** Immunodeficiencies are a heterogeneous group of disorders classified etiologically as primary (congenital) or secondary (acquired). Primary immunodeficiencies (PIDs), such as common variable immunodeficiency (CVID), result from genetic mutations that impair the development and function of lymphocytes. Secondary immunodeficiencies (SIDs) arise as a consequence of chronic diseases, lymphoid malignancies, or immunosuppressive therapies. **Aim of the study:** The purpose of this study was to assess the serum expression profile of selected microRNAs (miRNAs) in patients with CVID and in those with chronic lymphocytic leukemia (CLL) and coexisting SID, compared to healthy individuals. **Methods**: Digital PCR (dPCR) was applied to quantify the serum expression levels of selected miRNAs in patients with CVID, patients with CLL and SID, and in healthy controls. **Results:** dPCR revealed significantly reduced levels of miR-16, miR-30c, miR-181a, miR-29a, miR-150, and miR-326 in the CVID group, potentially reflecting impaired regulatory mechanisms of the immune system. In contrast, elevated levels of miR-21, miR-125b, and miR-155 were observed in the CLL group with SID, suggesting their role in tumorigenesis and secondary immunosuppression. Correlations between miRNA levels and the expression of immune checkpoints (PD-1, CTLA-4, CD200) indicated the involvement of a complex regulatory network encompassing both humoral and cellular immune mechanisms. **Conclusions:** The results provide preliminary evidence that selected miRNAs could reflect disease-specific immune dysregulation patterns and may hold potential as diagnostic and prognostic biomarkers in both PIDs and SIDs.

## 1. Introduction

Immunodeficiencies are a heterogeneous group of disorders involving impaired immune system function, classified into primary (PIDs) and secondary (SIDs) based on etiology [1,2,3]. PIDs result from genetic defects affecting immune cell development or function, whereas SIDs arise due to chronic diseases, hematological malignancies, immunosuppressive therapy, or persistent infections.

Common Variable Immunodeficiency (CVID) is the most prevalent symptomatic PID, characterized by hypogammaglobulinemia, impaired antigen response, and variable T- and B-cell dysfunction. It affects 1 in 25,000 to 1 in 50,000 individuals, with onset usually occurring in adulthood [4,5]. Although mutations in genes such as TNFRSF13B, ICOS, and CD19 have been identified, the pathogenesis remains incompletely understood and likely multifactorial. It is also recognized that CVID may predispose to hematological malignancies—including lymphoma and, more rarely, leukemia—as a consequence of chronic immune activation, impaired immune surveillance, and cumulative genomic instability [5]. A well-described form of SID occurs in chronic lymphocytic leukemia (CLL), a clonal B-cell malignancy associated with secondary immune dysfunction in over 80% of patients. This includes hypogammaglobulinemia, T/NK cell impairment, and increased susceptibility to infections and viral reactivation (e.g., EBV) [6,7]. Although CVID and CLL differ in etiology, both are associated with similar immune phenotypes, including disrupted immunoglobulin production, immune cell dysregulation, and altered expression of checkpoint molecules. This functional overlap raises the possibility that shared or parallel molecular mechanisms, particularly involving regulatory non-coding RNAs, may underlie immune impairment in both settings.

In recent years, microRNAs (miRNAs) have been shown to play crucial roles in regulating immune homeostasis, immune cell differentiation, and controlling the inflammatory response. Molecules such as miR-155, miR-150, and miR-125b have been extensively described as key modulators of immune processes, including T- and B-cell activation, cytokine response, and immune tolerance. Dysregulation of their expression can lead to excessive inflammation activation, impaired central and peripheral tolerance, as well as attenuation of antitumor surveillance mechanisms [8,9,10]. Research conducted by scientists has shown that pathology within miRNAs involved in hematopoiesis, immune reactions, or carcinogenesis may significantly correlate with most disorders associated with immunodeficiencies [4]. The analysis of available scientific literature conducted in the PubMed database revealed only 111 papers on the role of miRNAs in the course of PID, of which only 6 analyzed the participation of these molecules in the pathogenesis of CVID. In studies on CVID to date, the most frequently analyzed microRNA molecules have been: miR-34a-5p, miR-125b-5p, miR-142-3p, miR-155-5p, miR-210, and miR-142 and miR-155 (in the context of non-infectious complications) [4,11,12,13,14,15]. According to the researchers, it is their disturbed expression that may affect the regulation of the inflammatory response, the differentiation of B lymphocytes, and the modulation of signaling pathways crucial for immune system homeostasis.

In the case of CLL, the PubMed database reports 436 publications, of which only two directly address the issue of SID in the context of this disease [16,17]. In CLL, microRNAs modulate both leukemic cell survival and proliferation as well as the tumor microenvironment. The expression of miR-155 and miR-34a is significantly increased in CLL cells, affecting the expression of cell cycle regulatory proteins, costimulatory receptors, and immune checkpoints, such as PD-1 (programmed death receptor 1) and CTLA-4 (cytotoxic T-lymphocyte-associated antigen-4), which may lead to immunosuppression and tumor escape from the immune system. Thus, miRNAs play an important role in regulating the expression of genes responsible for the immune checkpoints, which are the fundamental mechanism for balancing the activation and inhibition of T and B lymphocyte responses, thereby conditioning immune tolerance and preventing their overactivation and functional exhaustion [18,19,20,21]. Disturbances in the expression levels of selected microRNAs can lead to deregulation of molecular pathways involving immune checkpoint molecules such as PD-1, PD-L1, and CTLA-4. This leads to abnormalities in lymphocyte activation, impaired immune tolerance mechanisms, and the development of chronic immune exhaustion. Such changes contribute to the development of a dysfunctional lymphocyte phenotype, observed in various diseases, including cancer and autoimmune diseases [16,22,23]. Despite growing interest in the regulatory role of miRNAs in immunology, the relationship between circulating microRNAs and immune exhaustion pathways has not yet been systematically investigated in patients with PID or SIDs, including CVID and CLL with SID.

In light of the above, we hypothesized that the deregulation of specific circulating miRNAs may reflect distinct mechanisms of immunoregulatory dysfunction in PID and SID. In CVID, the miRNA profile may indicate intrinsic defects in lymphocyte differentiation, dysfunctional germinal centers in lymph nodes, and impaired peripheral tolerance mechanisms, leading to chronic immune activation and the development of autoimmunity. MicroRNAs such as miR-155, miR-181a, and miR-150, which are known for their roles in regulating B-cell maturation and TCR signaling, may be involved in the loss of control over immune homeostasis. In CLL with SID, however, abnormal miRNA expression is likely secondary to the effects of the tumor microenvironment. In this situation, miRNA expression reflects tumor-induced immunosuppression, chronic antigenic stimulation, and the development of effector cell exhaustion. Deregulated miRNAs may promote the expression of checkpoints such as PD-1, CTLA-4, and CD200R, while simultaneously inhibiting the cytotoxic functions of T and NK cells. Specific miRNAs secreted by leukemia cells or transported in exosomes, such as miR-21, miR-29b, and miR-34a, influence the suppression of transcriptional programs responsible for activating the antitumor response, strengthening the immunosuppressive feedback loop.

We further hypothesize that correlations between serum miRNA profiles and checkpoint expression (PD-1, PD-L1, CTLA-4, CD200, CD200R) may differ significantly between CVID and CLL with SID. These differences may reveal both common and individual-specific regulatory networks driving immune dysfunction in both conditions. These differences stem from distinct pathogenetic backgrounds—inherited and developmental immunoregulatory defects predominate in CVID, whereas secondary, acquired, tumor-induced immune reprogramming is observed in CLL with SID.

From a clinical perspective, comparing PID (CVID) and SID (CLL) is crucial. Both diseases manifest as a deficiency in the humoral immune response and increased susceptibility to infection, yet their etiopathogenesis and therapeutic implications are distinct. Understanding the interplay between miRNAs and immune checkpoints may not only enable better differentiation between types of immune deficiencies but also contribute to the identification of new prognostic and predictive biomarkers—reflecting the degree of immune exhaustion, risk of infection, or response to immunomodulatory therapy. Furthermore, understanding these mechanisms may open the door to developing therapeutic strategies aimed at restoring immune balance by modulating miRNA expression, inhibiting checkpoints, or individualizing treatment with immunoglobulins and checkpoint inhibitors based on the patient’s immune profile with PID or SID. To explore this hypothesis, we conducted a comparative molecular and phenotypic analysis in patients with CVID, patients with newly diagnosed and treatment-naive CLL with SID, and healthy controls. The inclusion of untreated patients was critical to eliminate potential confounding effects of prior therapies—particularly immunoglobulin replacement or chemotherapy—on miRNA levels or immune checkpoint expression. We integrated high-resolution serum miRNA profiling using digital PCR (dPCR) with multiparametric flow cytometric assessment of immune checkpoint molecules on T and B lymphocyte subpopulations. This combined approach was designed to identify disease-specific immunoregulatory signatures and to investigate the potential of circulating miRNAs as non-invasive biomarkers differentiating primary and secondary immunodeficiency states.

## 2. Materials and Methods

### 2.1. Patient Population Included in the Study

A total of 71 people participated in this study, classified into three groups: 26 patients diagnosed with common variable immunodeficiency (CVID), 30 patients with chronic lymphocytic leukemia (CLL) and coexisting secondary immunodeficiency (SID), and 15 healthy volunteers (HVs), who served as the control group. Participants were recruited prospectively during outpatient immunological consultations at an academic center, and a specialist in clinical immunology determined study eligibility based on strictly defined inclusion and exclusion criteria.

The inclusion criteria for the study were as follows: 18 years of age, an expected life expectancy of at least 12 months, no immunosuppressive therapy within the three months preceding study inclusion, and informed written consent to participate. The exclusion criteria included: the presence of active infection (viral, bacterial or fungal), a history of solid organ transplantation or hematopoietic cell transplantation, diagnosis of a cancer other than CLL in the active phase, immunosuppressive treatment due to autoimmune diseases, pregnancy or lactation, participation in different clinical trials involving experimental drugs, metastases to the central nervous system and severe mental disorders that prevented informed involvement in the study. All patients with diagnosed CLL were in Rai stage 1 and Binet stage A. SID in patients with CLL was diagnosed according to current guidelines as a state of secondary impairment of the immune system function resulting from the primary disease. The diagnostic criteria included a reduced IgG level below 5 g/L, the presence of recurrent infections (≥8 episodes in the previous 12 months requiring antibiotic therapy), a lack of response to vaccination despite normal immunoglobulin levels, and impaired T-cell and antigen-presenting cell function [24]. For organizational reasons, data on the mutation status in the IGHV gene were not obtained.

The diagnosis of CVID was based on the diagnostic criteria developed by the European Society for Immunodeficiencies (ESID), including a significantly reduced level of IgG immunoglobulins, impaired post-vaccination response to protein and polysaccharide antigens, occurrence of clinical symptoms after the age of 4 years, and exclusion of secondary causes of hypogammaglobulinemia, such as lymphoproliferative neoplasms, nephrotic syndrome, presence of monoclonal protein, or exposure to immunosuppressive drugs [25].

Biological material was collected from each participant, including 10 mL of venous blood in EDTA tubes for immunophenotypic analysis, and 5 mL of blood in tubes without anticoagulant to obtain serum for the determination of selected microRNA levels and concentrations of soluble forms of checkpoint molecules. Samples were collected from the basilic vein using aseptic techniques. Samples for serum analyses were incubated at room temperature until completely clotted, then centrifuged at 1500× *g* for 10 min at 4 °C. The obtained serum was immediately separated and frozen at −80 °C until further analysis.

The Bioethics Committee approved the study protocol number. KE-0254/247/11/2023 for patients with CLL and KE-0254/76/04/2024 for patients with CVID.

### 2.2. Immunophenotyping

To evaluate the expression of selected immune checkpoint molecules, including PD-1, PD-L1, CTLA-4, CD200, and CD200R, multiparametric flow cytometric analysis was performed on blood samples. This study focused on three major lymphocyte subsets: CD3+CD4+ T helper cells, CD3CD8+ cytotoxic T cells, and CD3-CD19+B cells. Whole blood samples were incubated with a panel of fluorochrome-conjugated monoclonal anti-human antibodies specific for the following markers: CD45 FITC, CD3 BV510, CD4 BV650, CD8 BV605, CD19 BB700, PD-1 PE, PD-L1 APC, CTLA-4 PE, CD200 APC, and CD200R PE (all from BD Biosciences, Franklin Lakes, NJ, USA). To improve fluorochrome performance and minimize spectral overlap, BD Horizon™ Brilliant Stain Buffer (BD Biosciences) was included in each staining reaction. After a 20 min incubation at room temperature in the dark, erythrocytes were lysed using BD FACS Lysing Solution (BD Biosciences) according to the manufacturer’s instructions. The remaining leukocytes were subsequently washed twice with BD Pharmingen™ Stain Buffer containing bovine serum albumin (BSA) to remove unbound antibodies and reduce nonspecific background staining. Analyses were performed on a CytoFLEX LX flow cytometer (Beckman Coulter, Indianapolis, IN, USA), equipped with daily quality control using CytoFLEX Daily QC Fluorosphere reagents to minimize instrument-related variability. Data were analyzed using Kaluza Analysis software version 2.1 (Beckman Coulter, Indianapolis, IN, USA). Data were analyzed using Kaluza Analysis Software version 2.1 (Beckman Coulter, Indianapolis, IN, USA). Initial gating was based on CD45 expression and FSC/SSC characteristics to isolate the lymphocyte population, followed by the discrimination and quantification of checkpoint molecule expression (Supplementary Materials, Figures S1 and S2).

### 2.3. miRNA Isolation from Patient Serum

MicroRNA (miRNA) isolation from serum was performed using the miRNeasy Serum/Plasma Advanced Kit (Qiagen Hilden, Germany) according to the manufacturer’s recommendations. A 200 µL sample was used for isolation, which was first lysed in the presence of 400 µL of QIAzol Lysis Reagent buffer, vigorously mixed for 1 min (vortexing). Then, the samples were incubated at room temperature for 5 min, after which 90 µL of chloroform was added, vigorously mixed for 15 s, and incubated again for 3 min. After extraction, the samples were centrifuged (12,000× *g*, 15 min, 4 °C) to separate the phases. The aqueous phase containing RNA was carefully transferred to a new tube and then mixed with 1.5 volumes of ethanol (96–100%). The prepared mixture was applied to the RNeasy Mini Spin Column, allowing RNA to bind to the membrane. The columns were washed successively with RWT and RPE buffers to remove protein and organic contaminants. RNA was eluted using 30 µL of RNase-free water after a short incubation and centrifugation. Finally, the purified RNA was stored at −80 °C until further analysis.

### 2.4. cDNA Synthesis (Reverse Transcription)

Reverse transcription of isolated RNA into complementary DNA (cDNA) was performed using the miRCURY LNA RT Kit (Qiagen) according to the manufacturer’s protocol. The reaction was prepared in a volume of 20 µL by mixing 10 µL of RNA with 2 µL of miRCURY RT Synth Primer Mix, 4 µL of miRCURY RT Reaction Buffer, and 4 µL of the enzymatic component miRCURY RT Enzyme Mix. The mixture was thoroughly mixed and then subjected to a reaction in a thermocycler under the following conditions: 60 min at 42 °C (reverse transcription) and subsequently 5 min at 95 °C (enzyme inactivation). The obtained cDNA was diluted 1:10 in RNase-free water and stored at −80 °C until used in quantitative analysis.

### 2.5. Quantitative Analysis of miRNA Using Digital PCR (dPCR)

Digital PCR (dPCR) technology was used to quantify the level of selected miRNA molecules using the QIAcuity EG PCR Kit (Qiagen) in combination with the EvaGreen intercalating dye. The reaction mixture contained: 4 µL of 3× EvaGreen PCR Master Mix, 1.2 µL of 10× primer mix (final primer concentration: 0.4 µM each), up to 5 µL of cDNA template, and RNase-free water—total reaction volume was 12 µL. The analysis used LNA primer and probe sets for the following microRNA molecules (Qiagen, with catalog numbers):

- hsa-miR-155-5p (YP02119311);
- hsa-miR-210-5p (YP02104321);
- hsa-miR-181a-5p (YP00206081);
- hsa-miR-134-5p (YP00205989);
- hsa-miR-125b-5p (YP00205713);
- hsa-miR-16-5p (YP002055702);
- hsa-miR-33a-5p (YP00205690);
- hsa-miR-30c-5p (YP00204783);
- hsa-miR-142-5p (YP00204722);
- hsa-miR-144-5p (YP00204670);
- hsa-miR-744-5p (YP00204663);
- hsa-miR-150-5p (YP00204660);
- hsa-miR-326 (YP00204512);
- hsa-miR-29a-5p (YP00204430);
- hsa-miR-28-5p (YP00204322);
- hsa-miR-21-5p (YP00204230);
- hsa-miR-15a-5p (YP00204066);
- hsa-miR-221-5p (YP00204032);
- hsa-miR-486-5p (YP00204001).

Samples were applied to a PCR plate, and then the content of each well was transferred to a special QIAcuity nanoplate. After sealing it with a dedicated membrane, amplification was performed in a thermocycler under the following conditions: polymerase activation at 95 °C for 2 min, then 40 cycles of denaturation (95 °C, 30 s) and hybridization and elongation (60 °C, 1 min), ending with a final incubation at 40 °C for 5 min. Data analysis was performed on the QIAcuity platform (Qiagen), enabling detection of the fluorescence signal generated by the EvaGreen dye binding double-stranded DNA. The final copy number of the miRNAs studied was expressed as the number of molecules per microliter of sample using QIAcuity Suite software, which provides automatic recalculation based on the number of positive nanoreactions.

### 2.6. Determination of Soluble Checkpoint Molecules

The serum concentrations of selected soluble immune checkpoint molecules and their respective ligands, including soluble forms of PD-1, PD-L1, CTLA-4, CD200, and CD200R, were quantified using enzyme-linked immunosorbent assay (ELISA). Analyses were conducted on previously collected and properly stored serum samples, following standardized procedures to ensure preanalytical consistency. Commercially available ELISA kits with validated analytical sensitivities were employed following the manufacturers’ protocols. The detection thresholds for the respective analyzes were as follows: sPD-1 (1.14 pg/mL; Invitrogen, Waltham, MA, USA), sPD-L1 (0.6 pg/mL; Invitrogen), sCTLA-4 (0.13 ng/mL; Invitrogen), sCD200 (20 pg/mL; Invitrogen), and sCD200R (11.89 pg/mL; Abcam, Cambridge, UK). Absorbance readings were obtained using a Victor 3 microplate reader (PerkinElmer, Waltham, MA, USA).

### 2.7. Statistical Analysis

Statistical analyses were conducted using Statistica version 13.3 (TIBCO Software Inc., Palo Alto, CA, USA) and GraphPad Prism version 10.5 (GraphPad Software, San Diego, CA, USA). The normality of data distribution was verified using the Shapiro–Wilk test. For variables that did not follow a normal distribution, nonparametric statistical methods were applied. Specifically, differences among multiple groups were analyzed using the Kruskal–Wallis test, followed by Dunn’s multiple comparison post hoc test to identify pairwise differences between specific groups. Relationships between quantitative variables were assessed using Spearman’s rank correlation coefficient (ρ). All statistical tests were two-tailed, and a *p*-value < 0.05 was considered statistically significant.

## 3. Results

### 3.1. Assessment of Clinical, Hematological, and Immunological Parameters of the Studied Groups, Including the Percentage of Immune Checkpoints on T and B Lymphocytes and Their Soluble Forms in Serum

The analysis included 71 participants, divided into three groups: 26 patients with CVID, 30 patients with CLL with SID, and 15 healthy volunteers (HVs), who formed the control group. A set of hematological parameters, immunoglobulin concentrations, and basic immunophenotypic features of peripheral blood lymphocytes was assessed. Statistical comparisons were performed among all groups in a three-way system (CVID vs. HV, CVID vs. SID, and SID vs. HV), and the results were presented as medians with interquartile ranges (Q1–Q3).

As expected, patients with CLL and coexisting SID showed significantly elevated total white blood cell and lymphocyte counts compared to both CVID and HV groups (*p* < 0.001), consistent with clonal B-cell proliferation. In contrast, CVID patients exhibited significant lymphopenia (*p* = 0.0015 vs. HV), reflecting impaired lymphocyte development or survival, hallmarks of PID. Monocyte and neutrophil counts were increased in the CLL group (*p* < 0.001), while neutrophils were significantly reduced in CVID compared to controls (*p* < 0.001), suggesting divergent patterns of innate immune involvement. Although eosinophil and basophil levels showed minor variation, a statistically significant reduction in eosinophils was noted in the CLL group versus HV (*p* = 0.011), though without clear clinical relevance. Both CVID and CLL patients presented with significantly reduced red blood cell counts and hemoglobin levels compared to HV (*p* < 0.001), consistent with anemia of chronic disease or bone marrow suppression. Serum immunoglobulin profiles revealed marked reductions in IgG and IgA levels in both CVID and CLL patients relative to healthy controls (*p* < 0.001). Notably, IgG levels were significantly lower in CVID than in CLL (*p* < 0.001), reinforcing the more profound humoral defect in primary immunodeficiency (Table 1).

In terms of immunophenotype, the percentage of CD3+ T cells was significantly decreased in CLL with SID compared to both CVID and HVs (*p* < 0.001). In contrast, CD19+ B cells were markedly expanded in CLL (*p* < 0.001), consistent with neoplastic B-cell accumulation. CLL with SID patients also demonstrated significantly reduced percentages of CD4+ and CD8+ T cells, indicating a global dysfunction of T cells. In contrast, CVID patients displayed relatively preserved T-cell subset distribution despite total lymphopenia. The CD4+/CD8+ ratio did not differ significantly between groups (Table 1).

**Figure 1:**
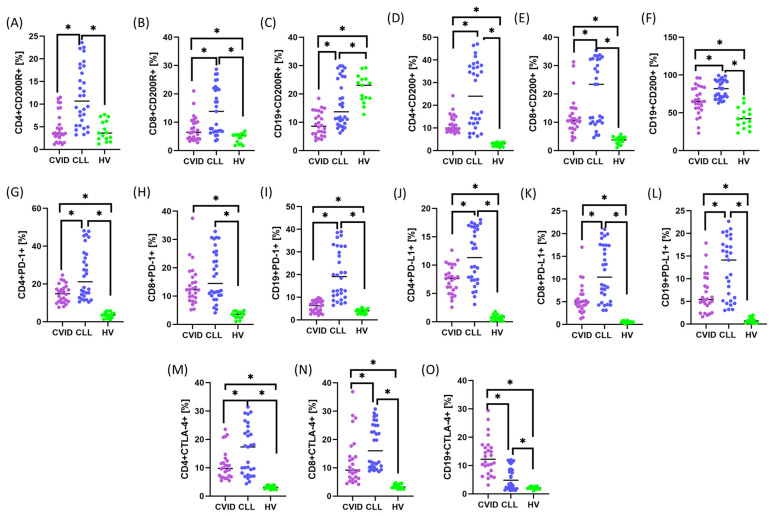
Expression of immune checkpoint molecules (CD200, PD-1, PD-L1, and CTLA-4) on T and B lymphocyte subpopulations in patients with CVID, CLL with SID, and healthy volunteers (HV). The graphs show the percentage of cells expressing individual molecules: (**A**) CD4+CD200R+, (**B**) CD8+CD200R+, (**C**) CD19+CD200R+, (**D**) CD4+CD200+, (**E**) CD8+CD200+, (**F**) CD19+CD200+, (**G**) CD4+PD-1+, (**H**) CD8+PD-1+, (**I**) CD19+PD-1+, (**J**) CD4+PD-L1+, (**K**) CD8+PD-L1+, (**L**) CD19+PD-L1+, (**M**) CD4+CTLA-4+, (**N**) CD8+CTLA-4+ and (**O**) CD19+CTLA-4+. Patients with common variable immunodeficiency (CVID), representing the primary immunodeficiency (PID) group, are shown in purple. Patients with chronic lymphocytic leukemia (CLL) and concomitant secondary immunodeficiency (SID) are depicted in blue, while healthy volunteers (HV), constituting the control group, are represented in green. Horizontal lines indicate median values, and asterisks (*) indicate statistically significant differences between the analyzed groups (*p* < 0.05).

Importantly, all patients were newly diagnosed and had not received any form of immunoglobulin replacement or immunosuppressive therapy before inclusion. This allowed for an unbiased evaluation of the immune phenotype and avoided the confounding effects of treatment. These comparative baseline data provide essential immunological context for the downstream investigation of immune checkpoint expression and miRNA regulatory signatures in the pathogenesis of immune dysfunction. The next stage of immunophenotypic analysis focused on evaluating the expression of key immune checkpoint molecules—PD-1, PD-L1, CTLA-4, CD200, and CD200R—on major T and B lymphocyte subsets. Deregulation of these pathways is known to contribute to immune exhaustion, impaired peripheral tolerance, and chronic immune activation. In patients with CVID and CLL with SID, a significantly increased expression of CD200 was observed on CD4+, CD8+, and CD19+ lymphocytes compared HV (*p* < 0.001), with the highest levels consistently found in CLL with SID (*p* < 0.001 vs. CVID for all subsets; Figure 1D–F). Conversely, expression of the inhibitory receptor CD200R on B cells (CD19+CD200R+) was significantly reduced in both patient groups relative to HV (*p* < 0.001), with CVID showing the lowest levels (*p* = 0.0489 vs. CLL with SID; Figure 1C). In contrast, CD200R expression on CD4+ and CD8+ T cells was significantly increased in CLL+SID patients compared to both CVID and HV (*p* < 0.001; Figure 1A,B), while no significant differences were found between CVID and HV for CD4+ cells (Supplementary Materials Table S1).

Checkpoint molecule CTLA-4 was markedly upregulated in all analyzed lymphocyte subsets (CD4+, CD8+, and CD19+) in both patient groups versus HV (*p* < 0.001), with the strongest expression observed again in CLL+SID (*p* = 0.048–<0.001 vs. CVID; Figure 1M–O). A similar trend was found for PD-1: its expression was significantly higher on CD4+, CD8+, and CD19+ lymphocytes in both patient groups relative to HV (*p* < 0.001), with CLL with SID demonstrating the highest PD-1 levels, particularly in the CD4+ subset (*p* = 0.003 vs. CVID; Figure 1G–I). Expression of PD-L1 followed this pattern, being elevated across all cell types in both CVID and CLL, with SID patients compared to controls (*p* < 0.001). The highest values were consistently found in the CLL with SID group (Figure 1J–L) (Supplementary Materials). The highest values were consistently found in the CLL with SID group (Figure 1J–L) (Supplementary Materials, Table S1).

**Figure 2:**
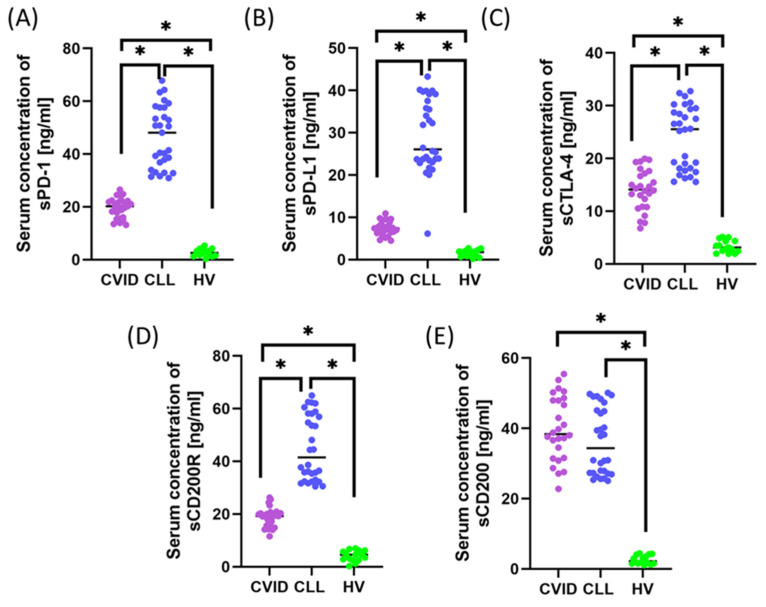
Serum concentrations of soluble forms of immune checkpoint molecules and their ligands in patients with CVID, CLL with SID, and healthy volunteers (HVs). Graphs show: (**A**) soluble PD-1 (sPD-1), (**B**) soluble PD-L1 (sPD-L1), (**C**) soluble CTLA-4 (sCTLA-4), (**D**) soluble CD200R receptor (sCD200R), and (**E**) soluble CD200 ligand (sCD200). Patients with common variable immunodeficiency (CVID), representing the primary immunodeficiency (PID) group, are shown in purple. Patients with chronic lymphocytic leukemia (CLL) and concomitant secondary immunodeficiency (SID) are depicted in blue, while healthy volunteers (HV), constituting the control group, are represented in green. Horizontal lines indicate median values, while asterisks (*) indicate statistically significant differences between the analyzed groups (*p* < 0.05).

When assessing soluble forms of checkpoints using ELISA, significantly higher levels of PD-1, PD-L1, CTLA-4, CD200, and CD200R were found in patients with CVID and CLL with SID compared to HVs (all *p* < 0.001). Additionally, PD-1, PD-L1, CTLA-4, and CD200R levels were significantly higher in CLL than in CVID. Only sCD200 levels did not differ considerably between CVID and CLL with SID (*p* = 0.134) (Supplementary Materials Table S1; Figure 2A–E).

### 3.2. Expression Profile of Selected microRNA Molecules in the Serum of CVID and CLL Patients with SID

**Figure 3:**
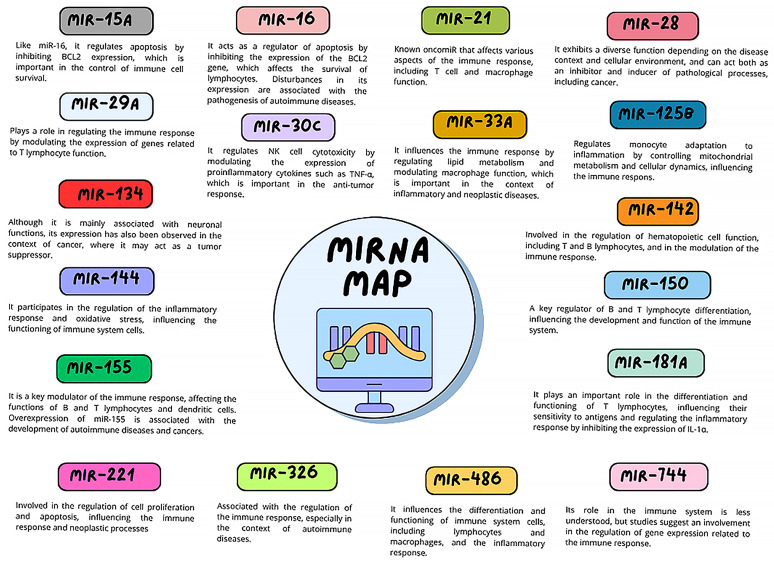
The figure summarizes the biological significance of selected miRNAs analyzed in the study (own work based on: [12,26,27,28,29,30,31,32,33,34,35,36,37,38,39,40,41,42,43,44,45]). The analyzed miRNAs include miR-142-3p and miR-155-5p [12,29], miR-15a/16 [28,31], miR-21 [27], miR-29a [26], miR-30c [33], miR-28 [35], miR-33a [42], miR-125b [43,45], miR-134 [40], miR-144 [36], miR-150 [34], miR-181a [37], miR-221/222 [32], miR-326 [30], miR-486 [39], miR-744-5p [38], miR-34a-5p [44], and miR-142 [41].

A further stage of this study involved quantitative analysis of selected miRNA molecules in the serum of recruited participants using digital polymerase chain reaction (dPCR). This technique was chosen as an analytical method due to its exceptional precision, sensitivity, and ability to provide absolute quantification of miRNA molecules in serum, eliminating the need for calibration curves. Compared to classic real-time PCR (qPCR), dPCR allows for the detection of low-level miRNA molecules, the concentrations of which in plasma or serum may be extremely low and subject to significant biological fluctuations. By dividing the reaction mixture into thousands of microscopic compartments (nanochambers), in which amplification occurs independently, it is possible to obtain high-resolution data with minimal influence of contaminants, reaction inhibitors, or differences in reverse transcription efficiency. This technique is also characterized by high repeatability and resistance to factors that interfere with the analysis, which is particularly important when examining biological materials with a complex matrix, such as serum. In the context of assessing the miRNA profile in patients with immunodeficiencies—both primary (CVID) and secondary (CLL with SID)—dPCR is therefore an optimal tool for detecting subtle changes in the expression of regulatory miRNAs, which may reflect complex immunological, neoplastic, or inflammatory phenomena. The choice of the miRNA panel for analysis in the study of patients with primary (CVID) and secondary (CLL with SID) immunodeficiencies was dictated by their documented roles in regulating the immune response, inflammatory processes, and neoplastic mechanisms, and is presented in Figure 3.

**Figure 4:**
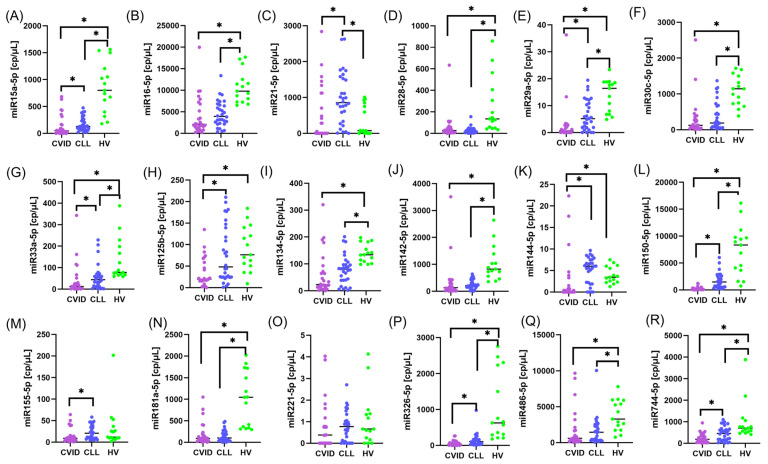
Serum expression of selected microRNAs in patients with CVID, CLL with secondary immunodeficiency (SID), and healthy volunteers (HV). Expression levels of 18 microRNAs were quantified by digital PCR (dPCR) and are presented as absolute values in copies per microliter (cp/µL). (**A**) miR-15a-5p, (**B**) miR-16-5p, (**C**) miR-21-5p, (**D**) miR-28-5p, (**E**) miR-29a-5p, (**F**) miR-30c-5p, (**G**) miR-33a-5p, (**H**) miR-125b-5p, (**I**) miR-134-5p, (**J**) miR-142-5p, (**K**) miR-144-5p, (**L**) miR-150-5p, (**M**) miR-155-5p, (**N**) miR-181a-5p, (**O**) miR-221-5p, (**P**) miR-326-5p, (**Q**) miR-486-5p, and (**R**) miR-744-5p. Each dot represents an individual subject, and horizontal bars indicate the median values. CVID patients are marked in purple as representatives of primary immunodeficiency (PID), CLL patients are marked in blue as representatives of secondary immunodeficiency (SID), and healthy volunteers are marked in green as the control group. Asterisks (*) indicate statistically significant differences between the analyzed groups.

The most pronounced decrease in expression compared to the control group was observed in the CVID group for miRNAs such as miR-15a (Figure 4A), miR-16 (Figure 4B), miR-28 (Figure 4D), miR-29a (Figure 4E), miR-30c (Figure 4F), miR-33a (Figure 4G), miR-125b (Figure 4H), miR-134 (Figure 4I), miR-142 (Figure 4J), miR-150 (Figure 4L), miR-181a (Figure 4N), miR-326 (Figure 4P), miR-486 (Figure 4Q) and miR-744 (Figure 4R) (all comparisons CVID vs. HV: *p* < 0.001). This applies primarily to microRNAs with known suppressive or regulatory effects on the control of lymphocyte differentiation and proliferation (Supplementary Materials, Table S2).

In the comparison of CLL with SID vs. HVs, significant differences were shown for most miRNAs, except, among others, miR-155 (Figure 4M) and miR-221 (Figure 4O), for which the levels did not differ significantly (*p* = 0.896 and *p* = 0.821). This means that the expression of these microRNAs may not be sufficiently sensitive in distinguishing the active phenotype of CLL with immunosuppression from that of a healthy immune system. However, it is worth noting that miR-155 reached statistical significance in the comparison of CVID versus CLL with SID (*p* = 0.010), indicating its potential importance in differentiating between inflammatory and neoplastic conditions (Supplementary Materials Table S2).

A comparison of CVID versus CLL with SID revealed fewer statistically significant differences than those observed in comparisons with the healthy group. Differences reaching the level of statistical significance (*p* < 0.05) were found for miR-15a (Figure 4A), miR-21 (Figure 4C), miR-29a (Figure 4E), miR-33a (Figure 4G), miR-125b (Figure 4H), miR-150 (Figure 4L), miR-326 (Figure 4P), and miR-744 (Figure 4R), which may indicate differential transcriptional regulation between primary (CVID) and secondary (CLL) immunodeficiency (Supplementary Materials Table S2).

This comparison reveals distinct molecular signatures between PID and SID, with a significant contribution from miRNAs, which may serve as potential biomarkers of immune dysregulation. The expression profile of molecules such as miR-16, miR-30c, miR-150, miR-125b, or miR-21 may reflect both the activation and attenuation of specific regulatory pathways in immunocompetent cells. It may also serve as a starting point for further studies on the diagnostic and prognostic applications of these findings.

### 3.3. Correlation Analysis Between Immune Parameters, Checkpoints, and miRNA Expression

To better understand the relationships between individual components of the immune response, a correlation analysis was performed, including peripheral blood morphological parameters, immunoglobulin levels, expression and concentration of immune checkpoints, and serum concentrations of selected miRNAs. This analysis aimed to identify associations between molecular regulators of the immune response (miRNA) and phenotypic features of the immune system, which may reflect the direction of exhaustion or immunosuppression in the course of primary and secondary immunodeficiencies.

**Figure 5:**
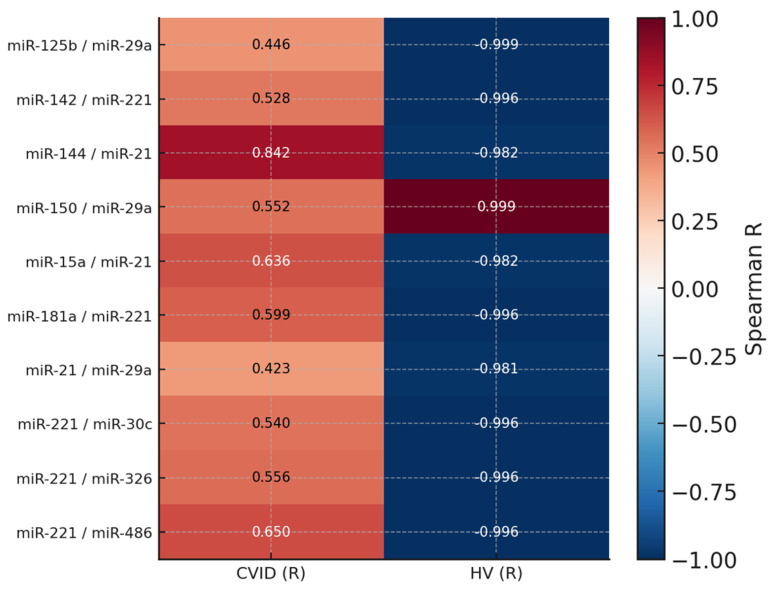
Heat map showing Spearman’s correlation coefficients (R) between selected circulating miRNA pairs in patients with common variable immunodeficiency (CVID) and healthy volunteers (HV). Each square represents the correlation value for a given miRNA pair within the respective study group. Positive correlations are shown in red, and negative correlations in blue, with color intensity reflecting the strength of association (scale from −1 to 1).

In the case of CVID patients, 114 statistically significant correlations were noted, of which 16 were negative correlations (4 low, 11 moderate, and 1 high) and 98 were positive correlations (2 low, 69 moderate, 21 high, and 5 very high) (Supplementary Materials, Table S3). More than 2.6 times as many, or as many as 301, statistically significant correlations were observed in patients with CLL who had concomitant SID. Of these, 184 correlations were negative (31 low, 76 moderate, 74 high, and three very high), and the remaining 117 were positive correlations (19 low, 68 moderate, 25 high, and four very high) (Supplementary Materials Table S4). Among the control patients, only 84 correlations were statistically significant, of which 58 were negative (28 moderate and 30 complete) and 26 were positive (9 moderate and 17 complete) (Supplementary Materials, Table S5). The comparative analysis of statistically significant correlations for the three patient groups revealed distinct patterns of correlations characteristic of patients with CVID, CLL with secondary immunodeficiency, and healthy volunteers, which may reflect different pathophysiological mechanisms underlying primary and secondary immune disorders. In the first analyzed case, significant differences in the strength and direction of correlations of the studied pairs of molecules were demonstrated between patients with CVID and healthy individuals. Particular attention is drawn to the fact that the direction of correlation was reversed in several cases, which may indicate a disturbance of physiological regulatory mechanisms in patients with CVID. For example, a strong positive correlation between miR-144 and miR-21 observed in the CVID group (R = 0.84) changed to a strongly negative value in the control group (R = −0.98), suggesting a shift in the functional relationship between these molecules under conditions of immune dysregulation. A similar phenomenon was observed for the pairs miR-15a/miR-21, miR-125b/miR-29a, and miR-221 with miR-30c, miR-326, and miR-486. It is worth noting that miR-221, a molecule associated with proliferation, apoptosis, and inflammatory responses, showed a strong negative correlation with many immunoregulatory miRNAs under healthy conditions. In contrast, in CVID, the direction of correlation was positive (Figure 5).

**Figure 6:**
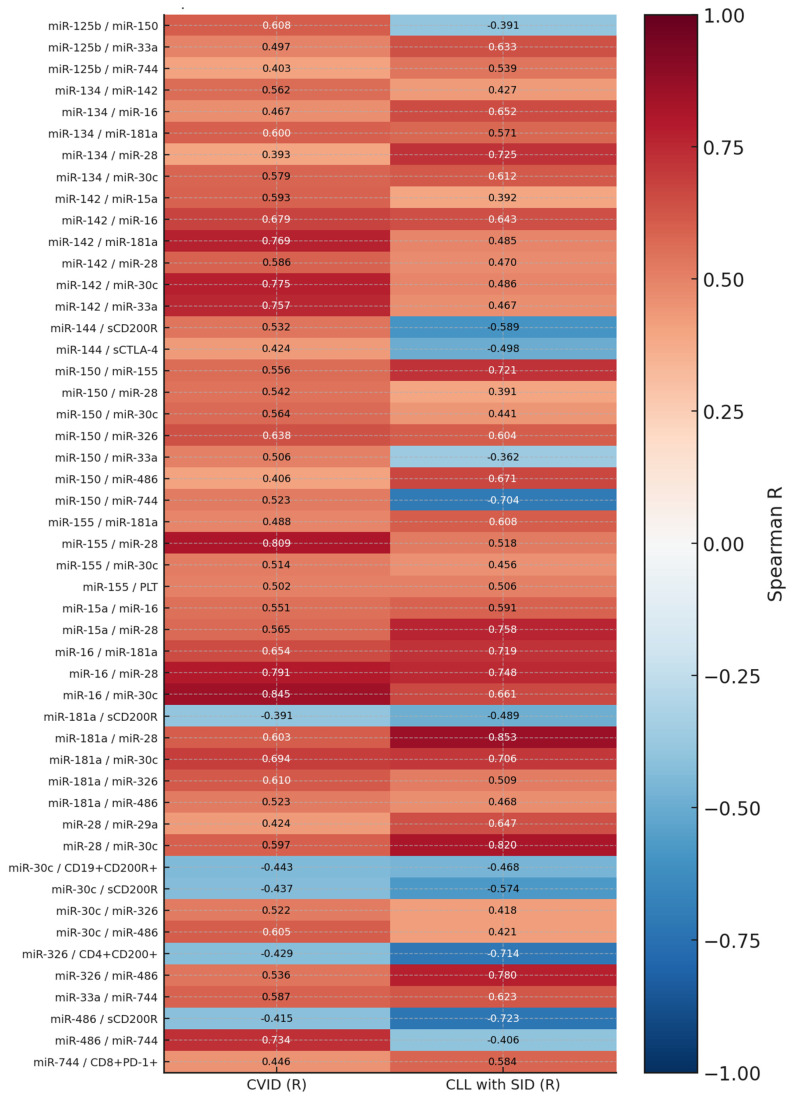
Heat map presenting Spearman’s correlation coefficients (*R*) for selected miRNA pairs and immune parameters in patients with common variable immunodeficiency (CVID) and chronic lymphocytic leukemia (CLL) with secondary immunodeficiency (SID). Each cell represents the strength and direction of correlation within the respective study group. Positive correlations are shown in red and negative correlations in blue, with color intensity corresponding to the magnitude of *R* (scale from −1 to 1).

In comparing correlations between CVID patients and CLL patients with coexisting SID, several significant differences were observed in the strength and direction of the relationships between selected miRNA molecules, as well as between miRNAs and immunological parameters. The most pronounced differences concerned pairs such as miR-125b and miR-150, miR-134 and miR-142, miR-142 and miR-16, miR-181a and miR-28, or miR-744 and miR-150, which in the CVID group showed strong positive correlations (R > 0.6), while in the CLL with SID group, they had low or even opposite (negative) values. Of particular interest is the observation of the reversal in the direction of correlation between the pair miR-150 and miR-744, where the positive correlation in CVID (R = 0.52) changed to a strongly negative one in CLL (R = −0.70). Additionally, miR-326 and CD4+CD200+ cells, as well as miR-486 and sCD200R, exhibited significant differences in correlation coefficient values, suggesting possible changes in pathways regulating the immune response that depend on the CD200/CD200R axis in different types of immunodeficiencies. Additionally, for pairs involving miR-181a (e.g., with miR-30c, miR-486, miR-28), both the strength and the number of positive correlations were higher in CLL than in CVID, which may suggest an increased activity of the pathways dependent on this miRNA in the context of cancer immune deregulation (Figure 6).

**Figure 7:**
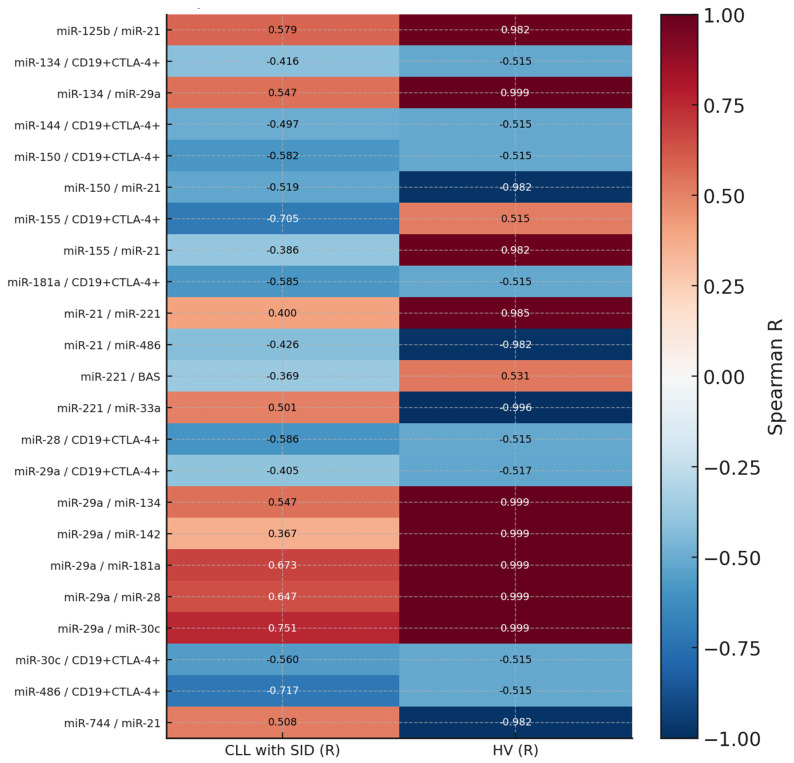
Heat map showing Spearman’s correlation coefficients (*R*) for selected circulating miRNAs and immune parameters in patients with chronic lymphocytic leukemia (CLL) with secondary immunodeficiency (SID) and healthy volunteers (HVs). Each cell represents the correlation strength and direction within the respective study group. Positive correlations are depicted in red, and negative correlations in blue, with color intensity indicating the magnitude of *R* (scale from −1 to 1).

In the comparative analysis of correlations between CLL patients with SID and healthy volunteers, numerous significant changes were noted in both the strength and direction of correlations of the studied parameters, including the expression of miRNA molecules and immune checkpoints (especially CTLA-4 on CD19+ B lymphocytes). Particularly significant were the reversals of correlations between miR-21 and selected miRNAs: a positive correlation between miR-125b and miR-21 (r = 0.579) in the SID group corresponded to an inverse, strongly negative relationship in the control group (r = −0.982). A similar phenomenon was also observed for the pairs miR-744 and miR-21, as well as miR-155 and miR-21, which may indicate a disturbance of physiological inflammatory and apoptotic surveillance in CLL patients. Additionally, numerous negative correlations were observed between CTLA-4 expression on B cells and immunoregulatory miRNAs such as miR-134, miR-144, miR-150, miR-181a, miR-28, miR-29a, miR-30c, and miR-486 (Figure 7).

**Figure 8:**
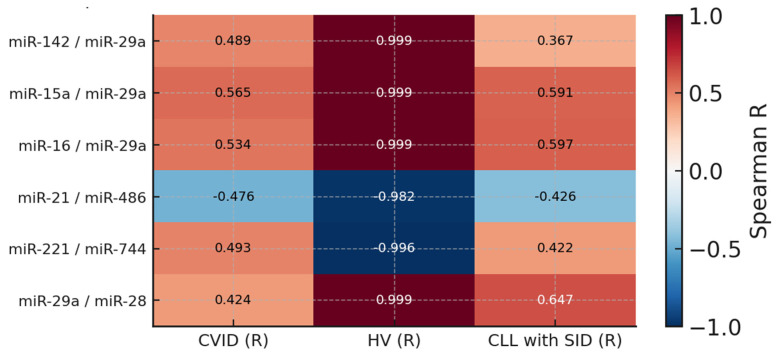
Heat map showing Spearman’s correlation coefficients (*R*) for common miRNA pairs shared across patients with common variable immunodeficiency (CVID), chronic lymphocytic leukemia (CLL) with secondary immunodeficiency (SID), and healthy volunteers (HVs). Each cell represents the correlation strength and direction within the respective group. Positive correlations are shown in red and negative correlations in blue, with color intensity reflecting the magnitude of *R* (scale from −1 to 1).

A cross-sectional analysis of selected microRNA pairs in the three studied groups revealed significant differences in the direction and strength of correlations, indicating variability in molecular regulation depending on the etiology of immune disorders. Apparent and consistent positive relationships were observed for pairs of miR-29a with miR-15a, miR-16, and miR-28, which remained within the range of positive correlations (r > 0.4) in all groups, with the highest values achieved in the control group and CLL with SID. This may suggest a relatively preserved co-regulation of these miRNAs in physiological and neoplastic conditions, and their weakening in CVID may result from a deeper defect in the primary differentiation of immune cells. The opposite trend was observed for the pair of miR-221 and miR-744, which showed a positive correlation in CVID (r = 0.493) and CLL (r = 0.422), whereas in the control group, it had a strongly negative value (r = −0.996). Similarly, miR-21 and miR-486 were negatively correlated in all groups, with the strongest correlation observed in the healthy population (r = −0.982). Their attenuation in CVID and CLL with SID may indicate a loss of control over apoptosis or inflammatory responses regulated by these molecules. The noticeable discrepancy in correlations may indicate a distinct profile of regulatory networks determined by genetic and immunological background, which is reflected in phenotypic and clinical differences between PID and SID (Figure 8).

## 4. Discussion

This study provides new insights into the differential expression patterns of circulating miRNAs in patients with CVID and CLL with SID, compared to HV. By focusing on serum-derived, cell-free miRNAs, our analysis captures systemic aspects of immune regulation rather than cell-specific events, which is particularly valuable when evaluating immunopathological states affecting multiple cell compartments.

We observed a broad downregulation of several miRNAs with well-established roles in lymphocyte development, immune tolerance, and the regulation of apoptosis. Notably, the expression of miR-142-3p and miR-155-5p was significantly reduced in CVID patients [12,46], consistent with prior experimental models showing that their deletion results in CVID-like phenotypes—hypogammaglobulinemia, lymphoproliferation, and autoimmunity [4]. Their dysregulation has also been associated with non-infectious complications, including lymphoma and autoimmune cytopenias [4].

A similar downregulation pattern was observed in both CVID and CLL with SID groups for miR-15a-5p and miR-29a-5p [15], which are known tumor suppressors involved in regulating BCL2 expression and lymphocyte survival. In the CLL cohort, this reduction likely reflects the underlying neoplastic process and aligns with well-documented 13q14 deletions affecting the miR-15a/16-1 cluster [47]. Reduced expression of miR-181a in CLL has also been linked to increased tumor aggressiveness and resistance to apoptosis through the PI3K/AKT pathway [47]. In contrast, overexpression of oncogenic and immunosuppressive miRNAs—miR-155-5p, miR-221-5p, and miR-144-5p—was observed exclusively in CLL patients with SID [15,48,49,50,51], suggesting their contribution to immune evasion via PD-L1 upregulation and T-cell dysfunction.

Interestingly, we also identified downregulation of miR-326-5p in both immunodeficient groups. Given its role in Th17 polarization and autoimmunity [52,53], this may indicate suppression of proinflammatory Th17 pathways. This finding corresponds well with the clinical presentation of recurrent infections and attenuated inflammation in CVID and advanced CLL.

Significantly, the observed miRNA expression profiles diverged between CVID and CLL with SID. While CVID was characterized predominantly by low levels of regulatory miRNAs (e.g., miR-142, miR-155), the CLL group showed a mixed signature: decreased suppressor miRNAs (miR-15a, miR-29a, miR-181a) alongside increased oncogenic/immunosuppressive ones (miR-155, miR-221). This contrast reflects the distinct underlying mechanisms of immune dysfunction—developmental failure in PID versus tumor-induced exhaustion in SID—and suggests that circulating miRNAs may serve as molecular markers to distinguish between these conditions. The results concerning miR-155 obtained in this study suggest that its expression level may have potential as a biomarker differentiating between primary (CVID) and secondary (CLL with SID) immunodeficiency. However, it is worth noting that the role of miR-155 in the pathogenesis of CLL has been previously described and involves a complex network of molecular interactions that contribute to the deregulation of B-cell signaling. In particular, as demonstrated in recent works, including the review by Maher et al. [54], mutations in the *XPO1* (exportin-1) gene lead to increased stability and expression of miR-155. Overexpression of this microRNA influences the B-cell receptor (BCR) signaling pathway, enhancing proproliferative and antiapoptotic signaling, which promotes the persistence of malignant B-cell populations. The described mechanism creates a molecular loop in which *XPO1* mutations enhance the expression of miR-155, which, in turn, through interaction with elements of the BCR pathway (including *SHIP1, PU.1*, and *IKKε*), further enhances signaling that promotes the survival of leukemic cells. In the context of our results, the differential expression of miR-155 between the CVID and CLL/SID groups may reflect these differences in the molecular mechanisms regulating B-cell activity. Considering this aspect highlights the importance of miR-155 not only as an immunoregulatory marker but also as a key element in the neoplastic transformation of B cells and the persistence of immunosuppression secondary to cancer [54].

miRNAs are key post-transcriptional regulators that modulate the expression of checkpoint receptors and ligands in both cancer cells and lymphocytes, as well as innate immune cells. Abnormalities in their levels promote T-cell exhaustion, deregulation of the effector response, and tumor escape from immune surveillance. Reviews emphasize that miRNAs can act in two ways: some inhibit IC expression (facilitating immune activation), while others stabilize or indirectly amplify inhibitory pathways, promoting immunosuppression and resistance to immunotherapy [16]. At the mechanistic level, numerous miRNAs directly target components of the PD-1/PD-L1 and CTLA-4 pathways, as well as their regulators. A systematic review (IJMS 2024) summarizes, among others, the miR-15/16 family (directing PD-1/PD-L1), let-7 (affecting PD-L1 glycosylation via the TCF-4/STT3 axis), and examples where administration of mimics/antagomirs modulated the efficacy of anti-PD-1/PD-L1/CTLA-4 antibodies in preclinical models. The same review also identifies miRNA profiles in peripheral leukocytes as potential biomarkers of response to checkpoint inhibitors (ICIs) [17,18]. In parallel, a comprehensive review in Frontiers in Immunology describes how miRNAs shape innate responses (e.g., via NK receptors, NKG2D, and its ligands), as well as specific responses (CD28 costimulation versus PD-1/CTLA-4 inhibitory signals), and apoptosis, which collectively determine the efficacy of immune surveillance and the course of ICI treatment. The authors provide functional examples: miR-28 regulates the T-cell “exhaustion” phenotype (affecting PD-1/TIM-3/LAG-3), miR-21 in macrophages modulates M1/M2 polarization. It may determine the efficacy of anti-PD-1 therapy, and miR-155 enhances T-cell responses by affecting the PI3K/AKT and STAT pathways [40]. Clinically, miRNAs are being investigated as predictors of ICI efficacy and as potential therapeutic targets (mimics/antagomirs)—alone or in combination with anti-PD-1/PD-L1/CTLA-4 antibodies. Reviews also highlight the importance of exosomal miRNAs as stable information carriers that can mediate immunosuppressive signals within the tumor microenvironment and serve as therapeutic vectors [40]. MiRNAs also play a crucial role in the pathogenesis of hematological malignancies, regulating several processes essential for the clonal transformation, growth, and survival of hematopoietic cells. In leukemias and lymphomas, dysregulation of miRNA expression can lead to disturbances in apoptosis, proliferation, differentiation, and migration of cancer cells. In many cases, the deregulation of oncogenic miRNAs (oncomiRs) and tumor suppressor miRNAs is observed, promoting the proliferation of tumor clones and resistance to treatment. An example is miR-155, whose overexpression has been demonstrated in various types of leukemias. It supports signaling pathways associated with proliferation (e.g., by affecting the PI3K/AKT and NF-κB pathways), suppresses the expression of tumor suppressor genes, and modulates the host immune response. Other miRNAs—such as miR-29, miR-15/16, or miR-34—have also been implicated in regulating oncogenic pathways and modulating the bone marrow microenvironment. Dysregulation of miRNAs may also promote immune escape by affecting immune checkpoints (e.g., by regulating PD-L1) or altering interactions between cancer cells and immune cells [55,56].

Despite growing evidence on miRNAs in hematological malignancies, studies investigating their role in primary immunodeficiencies remain scarce. To date, only a handful of reports have addressed serum miRNAs in CVID, and even fewer have compared them directly with SID-related immune defects [4,11,12,13]. Our study addresses this gap by providing a systematic, side-by-side molecular characterization of miRNA profiles in PID and SID. However, caution should be exercised when interpreting the data obtained. Avoiding overinterpretation is crucial due to the complexity of the regulatory networks involving miRNAs. The observed correlations and differences indicate potential biological mechanisms, but do not directly prove causal relationships. For example, although we found an association between the reduction of a given miRNA and the intensity of immunosuppression, we cannot clearly state whether the decrease in miRNA causes the impairment of immunity or is rather its consequence—a feedback loop and mutual reinforcement of these processes are likely. Moreover, specific miRNAs (e.g., miR-155, miR-150) are abundantly expressed within immune cells and can be released into the blood during their activation or death, which complicates the interpretation of serum levels. In the case of CLL, an additional factor is the expression of its own viral miRNAs by latently reactivating EBV, which may disrupt the host’s profiles [15]. It should be emphasized that the assessment of miRNA was performed only in serum, without parallel analysis of the expression of these molecules in immune cells (e.g., B and T lymphocytes, or monocytes). Although miRNAs circulating in serum may indirectly reflect immune activity and inflammation, their source remains ambiguous; they may originate from multiple cell types or be associated with the tumor, infectious, or inflammatory microenvironment. For this reason, the results do not permit a comprehensive assessment of the cellular mechanisms underlying the observed changes in expression.

### Study Limitations

The presented study has several significant limitations that should be taken into account when interpreting the results. First, the patient population analyzed was relatively small, which limited statistical power and the generalizability of the conclusions. The small sample size, particularly in patients with CLL with SID, increases the risk of bias due to individual biological variability. Due to the rarity of PID (such as CVID) and the difficulty in obtaining representative samples, the received data should be considered preliminary and require confirmation in larger cohorts.

Second, the CLL patient group was clinically homogeneous—all patients had stage I disease according to the Rai/Binet classification and were not undergoing anticancer treatment. This approach limited the influence of confounding factors such as immunosuppressive therapy or chemotherapy, but also prevented the generalization of results to later stages of disease progression or to patients undergoing treatment. It is therefore important to emphasize that the obtained results reflect early, potentially compensatory changes in microRNA profiles, which may undergo significant modifications as the disease progresses.

Another limitation is the lack of functional analyses that could confirm the biological significance of the observed correlations between microRNA expression and immune checkpoint molecules. The study was observational and cross-sectional in nature, which prevents assessment of the causal relationship between microRNA dysregulation and lymphocyte dysfunction. Changes in microRNA profiles over time or their potential predictive value for specific clinical events, such as infection rates, cancer progression, or treatment response, were also not analyzed.

Future prospective studies are desirable, encompassing larger and more diverse patient populations, which will allow for verification of the stability of the observed changes, their prognostic value, and their potential role in regulating the immune response. Supplementing the analyses with functional experiments—including assessment of microRNA target gene expression, in vitro analyses of checkpoint pathway modulation, and the impact of microRNAs on lymphocyte proliferation, apoptosis, and effector activity—would provide a better understanding of the mechanisms underlying the observed phenomena.

The identification of distinct serum miRNA expression patterns in these patients opens new avenues for research into the pathogenesis of immunodeficiencies. It supports the potential application of circulating miRNAs as non-invasive diagnostic and prognostic biomarkers in clinical immunology. This approach may facilitate the earlier detection of immune-related complications.

## 5. Conclusions

The present findings indicate that patients with CVID and CLL with SID display distinct serum expression patterns of selected microRNAs compared with healthy individuals. In the CVID group, lower expression levels of several microRNAs, including miR-16, miR-30c, miR-181a, miR-29a, miR-150, and miR-326, were observed, which may reflect alterations in immune regulation, chronic low-grade inflammation, or functional impairment of lymphocyte populations. Conversely, in patients with CLL and secondary immunodeficiency, elevated levels of miR-21, miR-125b, and miR-155 were detected, suggesting a potential relationship with tumor-associated immune dysregulation and secondary disturbance of immune homeostasis. The correlations identified between circulating miRNA levels and the expression of immune checkpoint molecules (PD-1, CD200, CTLA-4) in lymphocyte subsets may indicate the existence of an interconnected regulatory network integrating cellular and humoral immune responses.

The use of digital PCR (dPCR) allowed for accurate quantification of serum microRNA expression, supporting its feasibility as a methodological approach in immunological biomarker studies. However, the present results provide preliminary insights into the differential roles of microRNAs in PIDs and SIDs; further prospective and functional studies are required to confirm their biological relevance and clinical utility. Future research should aim to validate these findings in larger and more diverse cohorts, explore mechanistic links between miRNA regulation and immune checkpoint pathways, and assess the potential of circulating microRNAs as non-invasive biomarkers for disease monitoring and stratification in immunodeficiency disorders.

## Figures and Tables

**Table 1:** Hematological and immunophenotypic characteristics of patients with common variable immunodeficiency (CVID), chronic lymphocytic leukemia with secondary immunodeficiency (CLL with SID), and healthy volunteers (HV).

Parameters	CVID (PID)	CLL (SID)	HV	*p*-Value		
	Mediana (Q1–Q3)	Mediana (Q1–Q3)	Mediana (Q1–Q3)	CVID vs. HV	CVID vs. CLL	CLL vs. HV
WBC [10^3^/mm^3^]	6.54 (5.30–7.14)	30.91 (28.09–33.37)	5.94 (4.05–6.73)	0.120	<0.001 *	<0.001 *
LYM [10^3^/mm^3^]	0.74 (0.50–1.33)	26.85 (23.44–29.58)	1.69 (1.06–1.88)	0.0015 *	<0.001 *	<0.001 *
MON [10^3^/mm^3^]	0.48 (0.38–0.58)	0.64 (0.53–0.87)	0.46 (0.32–0.58)	0.925	<0.001 *	<0.001 *
NEU [10^3^/mm^3^]	1.12 (0.59–1.61)	2.49 (1.62–3.43)	2.66 (1.99–4.60)	<0.001 *	<0.001 *	0.022 *
EOS [10^3^/mm^3^]	0.08 (0.06–0.11)	0.07 (0.06–0.11)	0.09 (0.08–0.13)	0.400	0.631	0.011 *
BAS [10^3^/mm^3^]	0.03 (0.02–0.04)	0.03 (0.02–0.04)	0.03 (0.02–0.03)	0.461	0.607	0.840
RBC [10^6^/mm^3^]	3.43 (3.23–3.52)	3.34 (2.97–4.22)	4.54 (4.25–4.73)	<0.001 *	0.929	<0.001 *
HGB [g/dL]	9.90 (9.52–10.27)	10.01 (9.04–10.96)	13.10 (12.70–13.70)	<0.001 *	0.826	<0.001 *
PLT [10^3^/mm^3^]	115.00 (104–134)	140 (130–166)	267.00 (226–302)	<0.001 *	<0.001 *	<0.001 *
IgG [g/L]	4.94 (4.43–5.76)	6.27 (5.32–7.30)	11.30 (9.96–14.70)	<0.001 *	<0.001 *	<0.001 *
IgM [g/L]	1.20 (0.85–1.52)	1.10 (0.66–1.74)	1.30 (0.85–2.10)	0.400	0.903	0.677
IgA [g/L]	0.62 (0.37–0.75)	0.51 (0.27–0.78)	1.90 (1.60–2.62)	<0.001 *	0.382	<0.001 *
CD45+ [%]	91.70 (89.32–94.61)	93.57 (90.00–95.75)	93.63 (91.22–95.04)	0.165	0.438	0.712
CD3+ [%]	65.18 (59.42–73.09)	20.97 (16.96–27.150)	74.14 (69.12–84.63)	0.002 *	<0.001 *	<0.001 *
CD19+ [%]	8.62 (5.14–12.71)	60.74 (56.10–73.64)	48.08 (46.51–53.67)	<0.001 *	<0.001 *	<0.001 *
CD4+ [%]	30.24 (21.43–37.43)	11.11 (7.22–14.53)	39.06 (27.53–43.63)	0.149	<0.001 *	<0.001 *
CD8+ [%]	28.42 (22.06–42.41)	9.72 (6.24–16.06)	12.79 (8.22–13.88)	<0.001 *	<0.001 *	0.766
CD4+/CD8+ lymphocyte ratio	1.09 (0.66–1.70)	1.11 (0.59–1.69)	1.28 (1.16–1.95)	0.059	0.801	0.115

The following abbreviations were used throughout the table: CVID refers to Common Variable Immunodeficiency, which represents a primary immunodeficiency (PID). CLL stands for Chronic Lymphocytic Leukemia, and in this context, it is associated with SID, meaning Secondary Immunodeficiency. HV denotes Healthy Volunteers, who served as the control group in the study. Among hematological parameters, WBC indicates White Blood Cells, LYM refers to Lymphocytes, MON to Monocytes, NEU to Neutrophils, EOS to Eosinophils, BAS to Basophils, RBC stands for Red Blood Cells, HGB is Hemoglobin, and PLT represents Platelets. Immunoglobulin concentrations are indicated by IgG (Immunoglobulin G), IgA (Immunoglobulin A), and IgM (Immunoglobulin M). The immunophenotypic markers include CD45+ cells (pan-leukocyte marker), CD3+ T lymphocytes, CD19+ B lymphocytes, CD4+ helper T cells, and CD8+ cytotoxic T cells. Results marked with * are statistically significant (*p* < 0.05).

## Data Availability

Data supporting the study results can be made available by the corresponding author upon reasonable written request.

## Supplementary Materials

The following supporting information can be downloaded at https://www.mdpi.com/article/10.3390/cells14201577/s1, Figure S1: The figure illustrates the gating strategy used to analyze immune cell subpopulations by flow cytometry; Figure S2: The figure shows selected lymphocyte subpopulations studied by flow cytometry and the setting of cutoff points using FMO controls; Table S1: Comparison of immune checkpoint expression in lymphocyte subpopulations and concentration of soluble forms of regulatory molecules (sPD-1, sPD-L1, sCTLA-4, sCD200, sCD200R) in serum of patients with CVID (Common Variable Immunodeficiency), CLL with secondary immunodeficiency (Chronic Lymphocytic Leukemia with Secondary Immunodeficiency) and healthy volunteers (HV); Table S2: Expression of selected microRNAs in serum of patients with CVID (Common Variable Immunodeficiency), CLL with secondary immunodeficiency (Chronic Lymphocytic Leukemia with Secondary Immunodeficiency) and healthy volunteers (HV – Healthy Volunteers); Table S3: Correlation between the levels of selected microRNAs and the expression of immune checkpoints and clinical parameters in patients with CVID (Common Variable Immunodeficiency); Table S4: Correlations between microRNA levels and immune checkpoint expression in healthy volunteers (HV); Table S5: Correlations between microRNA levels and immune checkpoint expression in patients with chronic lymphocytic leukemia and secondary immunodeficiency (CLL with SID).

## Abbreviations

The following abbreviations are used in this manuscript:

CD	Cluster of Differentiation
CLL	Chronic Lymphocytic Leukemia
CVID	Common Variable Immunodeficiency
CTLA-4	Cytotoxic T-Lymphocyte-Associated Antigen-4
dPCR	Digital Polymerase Chain Reaction
EBV	Epstein–Barr Virus
ICOS	Inducible T-Cell Costimulator
miRNA (miR)	MicroRNA
NK	Natural Killer
PD-1	Programmed Death Receptor 1
PD-L1	Programmed Death-Ligand 1
PID	Primary Immunodeficiency
SID	Secondary Immunodeficiency
TNFRSF13B	Tumor Necrosis Factor Receptor Superfamily Member 13B
