# Supplementary material for: MicroRNA Deregulation and Immune Checkpoint Interactions in Common Variable Immunodeficiency and CLL-Associated Secondary Immunodeficiency
Source: Cells. 2025 Oct 10;14(20):1577. doi: 10.3390/cells14201577 (PMC12564553; doi:10.3390/cells14201577)
Supplement: Supplementary file 1 [file cells-14-01577-s001.zip › cells-3888211-supplementary.pdf]

Supplemenatry Materials Tabel S1: Comparison of immune checkpoint expression in lymphocyte subpopulations and concentration of soluble forms of regulatory molecules (sPD-1, sPD-L1, sCTLA-4, sCD200, sCD200R) in serum of patients with CVID (Common Variable Immunodeficiency), CLL with secondary immunodeficiency (Chronic Lymphocytic Leukemia with Secondary Immunodeficiency) and healthy volunteers (HV). Data are presented as medians and quartiles of the interquartile range (Q1–Q3). P values are given for intergroup comparisons (CVID vs. HV, CVID vs. CLL, CLL vs. HV); p < 0.05 was considered statistically significant (*).

Parameters	CVID	CLL (SID)	HV	CVID vs. HV	CVID vs. CLL	CLL vs. HV
	Median (Q1-Q3)	Median (Q1-Q3)	Median (Q1-Q3)			
CD19+CD200+ [%]	65.01 (56.21-82.14)	78.47 (70.65-92.41)	42.22 (31.93-54.43)	p<0.001 *	0.001*	p<0.001 *
CD4+CD200+ [%]	9.94 (8.69-14.02)	23.97 (12.08-37.24)	3.03 (1.85-3.48)	p<0.001 *	0.001*	p<0.001 *
CD8+CD200+ [%]	10.55 (7.90-14.85)	17.76 (9.75-32.25)	3.79 (2.55-4.68)	p<0.001 *	0.025*	p<0.001 *
CD19+CD200R+ [%]	8.57 (5.91-13.57)	13.68 (9.58-26.07)	23.06 (18.23-25.27)	p<0.001 *	p<0.001 *	0.0489
CD4+CD200R+ [%]	3.58 (2.64-7.04)	10.67 (5.25-17.99)	3.61 (2.36-6.24)	0.758407	p<0.001 *	p<0.001 *
CD8+CD200R+ [%]	6.44 (4.17-10.07)	11.54 (6.71-21.98)	5.34 (2.31-5.62)	0.012*	0.004	p<0.001 *
CD19+CTLA-4+ [%]	12.25 (8.21-16.33)	4.80 (1.88-8.73)	2.13 (1.90-2.55)	p<0.001 *	p<0.001 *	0.011517
CD4+CTLA-4+ [%]	9.76 (6.83-12.43)	14.62 (7.46-22.81)	3.10 (2.54-3.75)	p<0.001 *	0.0480	p<0.001 *
CD8+CTLA-4+ [%]	9.19 (6.25-14.01)	16.03 (10.29-24.99)	3.20 (2.93-4.26)	p<0.001 *	0.004	p<0.001 *
CD19+PD-1+ [%]	6.26 (3.74-8.22)	19.11 (10.68-32.48)	3.96 (2.65-4.70)	0.018*	p<0.001 *	p<0.001 *
CD4+PD-1+ [%]	14.81 (10.67-17.77)	21.10 (13.61-35.73)	3.54 (2.31-4.53)	p<0.001 *	0.003	p<0.001 *
CD8+PD-1+ [%]	12.24 (9.43-16.69)	14.45 (10.27-25.56)	3.63 (2.45-4.31)	p<0.001 *	0.223	p<0.001 *
CD19+PDL-1+ [%]	5.40 (4.09-10.09)	12.52 (5.14-17.59)	0.67 (0.28-1.40)	p<0.001 *	0.004	p<0.001 *
CD4+PD-L1+ [%]	7.63 (5.63-9.54)	11.33 (7.35-16.64)	0.73 (0.27-1.00)	p<0.001 *	0.003	p<0.001 *
CD8+PD-L1+ [%]	4.90 (3.92-6.63)	10.40 (5.06-17.45)	0.51 (0.37-0.70)	p<0.001 *	p<0.001 *	p<0.001 *
sPD-1 [ng/mL]	20.24 (16.04-22.21)	45.20 (36.86-57.60)	2.55 (1.55-4.10)	p<0.001 *	p<0.001 *	p<0.001 *
sPD-L1 [ng/mL]	7.35 (6.28-8.90)	26.06 (23.48-37.51)	1.75 (0.83-2.07)	p<0.001 *	p<0.001 *	p<0.001 *
sCTLA-4 [ng/mL]	14.15 (10.85-17.16)	25.55 (18.10-29.34)	3.12 (2.42-4.35)	p<0.001 *	p<0.001 *	p<0.001 *
sCD200 [ng/mL]	38.38 (31.56-47.95)	34.39 (27.28-45.12)	2.17 (1.50-4.08)	p<0.001 *	0.134	p<0.001 *
sCD200R [ng/mL]	19.25 (15.63-20.33)	41.52 (32.95-58.09)	4.57 (2.52-6.15)	p<0.001 *	p<0.001 *	p<0.001 *

Supplementary Materials Tabel S2: Expression of selected microRNAs in serum of patients with CVID (Common Variable Immunodeficiency), CLL with secondary immunodeficiency (Chronic Lymphocytic Leukemia with Secondary Immunodeficiency) and healthy volunteers (HV – Healthy Volunteers). Data are presented as copies per microliter (cp/µL) – median values and quartiles of the interquartile range (Q1–Q3). Statistical significance of intergroup comparisons (CVID vs. HV, CVID vs. CLL, CLL vs. HV) is presented; $p < 0.05$ was considered statistically significant (*).

Parameters	CVID	CLL (SID)	HV	CVID vs. HV	CVID vs. CLL	CLL vs. HV
	Median (Q1-Q3)	Median (Q1-Q3)	Median (Q1-Q3)			
miR16-5p [cp/µL]	2075.85 (1362.8-6800.3)	3920.79 (2305.87-6577.11)	9780.64 (7285.93-12520.23)	p<0.001 *	0.147	p<0.001 *
miR155-5p [cp/µL]	8.88 (4.06-14.05)	20.80 (9.36-38.35)	11.72 (9.65-36.80)	0.086	0.010*	0.896
miR30c-5p [cp/µL]	123.0 (50.22-356.5)	188.26 (83.17-661.09)	1144.46 (727.72-1497.81)	p<0.001 *	0.182	p<0.001 *
miR28-5p [cp/µL]	25.52 (8.81-55.99)	18.27 (7.54-37.82)	134.40 (48.48-406.58)	p<0.001 *	0.323	p<0.001 *
miR181a-5p [cp/µL]	90.16 (55.15-191.4)	100.84 (44.21-207.20)	1046.17 (334.37-1721.76)	p<0.001 *	0.877	p<0.001 *
miR134-5p [cp/µL]	22.83 (13.05-71.84)	79.71 (39.12-96.75)	133.82 (109.18-182.19)	p<0.001 *	0.051	p<0.001 *
miR125b-5p [cp/µL]	19.43 (15.07-58.64)	48.19 (24.54-139.07)	76.35 (50.66-139.65)	p<0.001 *	0.002*	0.277
miR33a-5p [cp/µL]	12.49 (2.26-30.36)	43.83 (12.67-70.11)	77.59 (67.29-198.09)	p<0.001 *	0.021*	p<0.001 *
miR142-5p [cp/µL]	132.25 (19.31-336.30)	209.79 (149.02-417.62)	821.93 (556.92-1387.33)	p<0.001 *	0.147	p<0.001 *
miR144-5p [cp/µL]	0.00 (0.00-1.495)	6.02 (2.21-7.57)	3.45 (2.33-6.03)	p<0.001 *	p<0.001*	0.176
miR744-5p [cp/µL]	186.15 (38.86-412.80)	455.08 (165.95-657.66)	702.27 (533.08-1054.46)	p<0.001 *	0.015*	0.007*
miR150-5p [cp/µL]	103.21 (45.71-270.90)	1471.90 (594.40-2599.90)	8337.29 (3665.89-9781.25)	p<0.001 *	p<0.001 *	p<0.001 *
miR326-5p [cp/µL]	36.92 (2.91-99.76)	105.43 (30.41-173.10)	626.40 (293.39-2239.69)	p<0.001 *	0.004*	p<0.001 *
miR29a-5p [cp/µL]	0.58 (0.00-2.70)	5.39 (1.87-12.10)	16.41 (6.76-18.77)	p<0.001 *	p<0.001*	p<0.001*
miR21-5p [cp/µL]	26.56 (0.00-1120.30)	856.17 (334.30-1525.44)	75.90 (0.00-849.55)	0.862	0.003*	0.002*
miR15a-5p [cp/µL]	48.43 (0.37-165.00)	128.15 (73.95-260.33)	800.11 (397-1206.13)	p<0.001 *	0.021*	p<0.001*
miR221-5p [cp/µL]	0.381 (0.00-0.783)	0.775 (0.08-1.05)	0.66 (0.16-1.37)	0.383	0.284	0.821
miR486-5p [cp/µL]	611.15 (229-1701.00)	1442.33 (313.38-2444.78)	3282.33 (1749.17-5905.25)	p<0.001 *	0.331	p<0.001 *

Supplementary Materials Table S3: Correlation between the levels of selected microRNAs and the expression of immune checkpoints and clinical parameters in patients with CVID (Common Variable Immunodeficiency). The analysis includes the relationships between the expression of serum miRNAs (expressed as cp/µL) and the percentage of lymphocytes expressing PD-1, PD-L1, CTLA-4, CD200, CD200R receptors and the concentrations of soluble forms of these molecules in serum (sPD-1, sPD-L1, sCTLA-4, sCD200, sCD200R). The values of correlation coefficients were calculated using the Spearman test.

Pair of variables - CVID	R	t(N-2)	p
miR29a & NEU	-0.64	-4.07	0.00
miR16 & NEU	-0.53	-3.03	0.01
miR30c & EOS	-0.52	-2.94	0.01
miR21 & miR486	-0.48	-2.65	0.01
miR221 & sPD1	-0.48	-2.65	0.01
miR29a & RBC	-0.46	-2.57	0.02
miR30c & CD19+CD200R+ [%]	-0.44	-2.42	0.02
miR16 & EOS	-0.44	-2.40	0.02
miR30c & sCD200R	-0.44	-2.38	0.03
miR326 & CD4+CD200+ [%]	-0.43	-2.33	0.03
miR181a & EOS	-0.42	-2.27	0.03
miR486 & sCD200R	-0.41	-2.23	0.04
miR15a & NEU	-0.40	-2.17	0.04
miR181a & sCD200R	-0.39	-2.08	0.05
miR221 & sCD200R	-0.39	-2.07	0.05
miR134 & miR28	0.39	2.09	0.05
miR134 & miR486	0.40	2.11	0.05
miR125b & miR744	0.40	2.16	0.04
miR142 & miR155	0.41	2.18	0.04
miR150 & miR486	0.41	2.18	0.04
miR155 & CD4+PDL-1+ [%]	0.42	2.25	0.03
miR125b & miR326	0.42	2.25	0.03
miR21 & miR29a	0.42	2.29	0.03
miR28 & miR29a	0.42	2.29	0.03
miR144 & sCTLA4	0.42	2.29	0.03
miR125b & miR15a	0.42	2.30	0.03
miR16 & miR744	0.43	2.31	0.03
miR125b & miR155	0.43	2.34	0.03
miR15a & miR33a	0.43	2.35	0.03
miR221 & CD19+CD200+ [%]	0.43	2.37	0.03
miR150 & miR15a	0.44	2.37	0.03
miR16 & miR486	0.44	2.40	0.02
miR15a & miR326	0.44	2.42	0.02
miR125b & miR29a	0.45	2.44	0.02
miR744 & CD8+PD-1+ [%]	0.45	2.44	0.02
miR134 & miR155	0.46	2.53	0.02
miR134 & IgG [g/L]	0.46	2.55	0.02
miR125b & miR134	0.46	2.56	0.02
miR21 & sCD200R	0.46	2.56	0.02
miR134 & miR16	0.47	2.58	0.02
miR125b & CD4+PDL-1+ [%]	0.47	2.60	0.02
miR134 & CD8+PD-1+ [%]	0.48	2.67	0.01
miR155 & miR181a	0.49	2.74	0.01
miR142 & miR29a	0.49	2.74	0.01
miR221 & miR744	0.49	2.78	0.01
miR125b & miR181a	0.50	2.80	0.01
miR125b & miR33a	0.50	2.81	0.01
miR155 & PLT	0.50	2.84	0.01
miR150 & miR33a	0.51	2.87	0.01
miR125b & miR28	0.51	2.91	0.01
miR150 & miR16	0.51	2.91	0.01
miR155 & miR30c	0.51	2.93	0.01
miR134 & miR744	0.51	2.94	0.01
miR134 & miR150	0.52	2.99	0.01
miR30c & miR33a	0.52	2.99	0.01
miR30c & miR326	0.52	3.00	0.01
miR150 & miR744	0.52	3.01	0.01
miR181a & miR486	0.52	3.01	0.01
miR142 & miR221	0.53	3.05	0.01
miR144 & sCD200R	0.53	3.08	0.01
miR125b & miR16	0.53	3.09	0.01
miR181a & miR33a	0.53	3.09	0.00
miR16 & miR29a	0.53	3.09	0.00
miR326 & miR486	0.54	3.11	0.00
miR221 & miR30c	0.54	3.14	0.00
miR150 & miR28	0.54	3.16	0.00
miR15a & miR16	0.55	3.23	0.00
miR150 & miR29a	0.55	3.24	0.00
miR150 & miR155	0.56	3.28	0.00
miR221 & miR326	0.56	3.28	0.00
miR144 & miR15a	0.56	3.29	0.00
miR134 & miR142	0.56	3.33	0.00
miR150 & miR30c	0.56	3.35	0.00
miR15a & miR28	0.56	3.35	0.00
miR15a & miR29a	0.56	3.35	0.00
miR125b & miR30c	0.57	3.36	0.00
miR134 & miR30c	0.58	3.48	0.00
miR142 & miR486	0.58	3.51	0.00
miR142 & miR28	0.59	3.55	0.00
miR33a & miR744	0.59	3.55	0.00
miR142 & miR15a	0.59	3.61	0.00
miR30c & miR744	0.59	3.61	0.00
miR28 & miR30c	0.60	3.65	0.00
miR181a & miR221	0.60	3.67	0.00
miR134 & miR181a	0.60	3.67	0.00

miR181a & miR28	0.60	3.71	0.00
miR125b & miR142	0.60	3.71	0.00
miR30c & miR486	0.61	3.73	0.00
miR125b & miR150	0.61	3.75	0.00
miR181a & miR326	0.61	3.77	0.00
miR155 & miR16	0.63	3.99	0.00
miR15a & miR21	0.64	4.03	0.00
miR150 & miR326	0.64	4.05	0.00
miR221 & miR486	0.65	4.19	0.00
miR16 & miR181a	0.65	4.24	0.00
miR150 & miR181a	0.66	4.33	0.00
miR142 & miR16	0.68	4.53	0.00
miR326 & miR744	0.69	4.61	0.00
miR142 & miR150	0.69	4.69	0.00
miR150 & miR142	0.69	4.69	0.00
miR181a & miR30c	0.69	4.73	0.00
miR181a & miR744	0.71	4.91	0.00
miR142 & miR744	0.72	5.05	0.00
miR486 & miR744	0.73	5.29	0.00
miR142 & miR33a	0.76	5.68	0.00
miR142 & miR181a	0.77	5.89	0.00
miR142 & miR30c	0.78	6.01	0.00
miR16 & miR28	0.79	6.33	0.00
miR155 & miR28	0.81	6.73	0.00
miR142 & miR326	0.83	7.24	0.00
miR326 & miR33a	0.83	7.30	0.00
miR144 & miR21	0.84	7.64	0.00
miR16 & miR30c	0.85	7.76	0.00

Supplementary Materials Table S4: Correlations between microRNA levels and immune checkpoint expression in healthy volunteers (HV). Spearman correlation coefficients between serum concentrations of selected miRNAs (cp/µL) and percentages of immune cells expressing PD-1, PD-L1, CTLA-4, CD200, CD200R receptors and concentrations of their soluble forms (sPD-1, sPD-L1, sCTLA-4, sCD200, sCD200R) were presented.

Pair of variables - HV	R	t(N-2)	p
miR125b & miR29a	-1.00	-85.25	0.00
miR155 & miR29a	-1.00	-85.25	0.00
miR134 & miR221	-1.00	-37.99	0.00
miR142 & miR221	-1.00	-37.99	0.00
miR144 & miR221	-1.00	-37.99	0.00
miR150 & miR221	-1.00	-37.99	0.00
miR15a & miR221	-1.00	-37.99	0.00
miR16 & miR221	-1.00	-37.99	0.00
miR181a & miR221	-1.00	-37.99	0.00
miR221 & miR28	-1.00	-37.99	0.00
miR221 & miR30c	-1.00	-37.99	0.00
miR221 & miR326	-1.00	-37.99	0.00
miR221 & miR33a	-1.00	-37.99	0.00
miR221 & miR486	-1.00	-37.99	0.00
miR221 & miR744	-1.00	-37.99	0.00
miR221 & miR29a	-0.99	-34.62	0.00
miR134 & miR21	-0.98	-18.73	0.00
miR142 & miR21	-0.98	-18.73	0.00
miR144 & miR21	-0.98	-18.73	0.00
miR150 & miR21	-0.98	-18.73	0.00
miR15a & miR21	-0.98	-18.73	0.00
miR16 & miR21	-0.98	-18.73	0.00
miR181a & miR21	-0.98	-18.73	0.00
miR21 & miR28	-0.98	-18.73	0.00
miR21 & miR30c	-0.98	-18.73	0.00
miR21 & miR326	-0.98	-18.73	0.00
miR21 & miR33a	-0.98	-18.73	0.00
miR21 & miR486	-0.98	-18.73	0.00
miR21 & miR744	-0.98	-18.73	0.00
miR21 & miR29a	-0.98	-18.25	0.00
miR29a & BAS	-0.52	-2.19	0.05
miR29a & CD19+CTLA-4+ [%]	-0.52	-2.18	0.05
miR134 & MON	-0.52	-2.17	0.05
miR142 & MON	-0.52	-2.17	0.05
miR144 & MON	-0.52	-2.17	0.05
miR150 & MON	-0.52	-2.17	0.05
miR15a & MON	-0.52	-2.17	0.05
miR16 & MON	-0.52	-2.17	0.05
miR181a & MON	-0.52	-2.17	0.05
miR28 & MON	-0.52	-2.17	0.05
miR30c & MON	-0.52	-2.17	0.05
miR326 & MON	-0.52	-2.17	0.05
miR33a & MON	-0.52	-2.17	0.05
miR486 & MON	-0.52	-2.17	0.05
miR744 & MON	-0.52	-2.17	0.05
miR134 & CD19+CTLA-4+ [%]	-0.51	-2.16	0.05
miR142 & CD19+CTLA-4+ [%]	-0.51	-2.16	0.05
miR144 & CD19+CTLA-4+ [%]	-0.51	-2.16	0.05
miR150 & CD19+CTLA-4+ [%]	-0.51	-2.16	0.05
miR15a & CD19+CTLA-4+ [%]	-0.51	-2.16	0.05
miR16 & CD19+CTLA-4+ [%]	-0.51	-2.16	0.05
miR181a & CD19+CTLA-4+ [%]	-0.51	-2.16	0.05
miR28 & CD19+CTLA-4+ [%]	-0.51	-2.16	0.05
miR30c & CD19+CTLA-4+ [%]	-0.51	-2.16	0.05
miR326 & CD19+CTLA-4+ [%]	-0.51	-2.16	0.05
miR33a & CD19+CTLA-4+ [%]	-0.51	-2.16	0.05
miR486 & CD19+CTLA-4+ [%]	-0.51	-2.16	0.05
miR744 & CD19+CTLA-4+ [%]	-0.51	-2.16	0.05

miR125b & CD19+CTLA-4+ [%]	0.51	2.16	0.05
miR155 & CD19+CTLA-4+ [%]	0.51	2.16	0.05
miR125b & MON	0.52	2.17	0.05
miR155 & MON	0.52	2.17	0.05
miR221 & MON	0.53	2.24	0.04
miR21 & BAS	0.53	2.24	0.04
miR221 & BAS	0.53	2.26	0.04
miR21 & CD8+[%]	0.55	2.35	0.04
miR221 & CD19+CTLA-4+ [%]	0.55	2.37	0.03
miR125b & miR21	0.98	18.73	0.00
miR155 & miR21	0.98	18.73	0.00
miR125b & miR221	1.00	37.99	0.00
miR155 & miR221	1.00	37.99	0.00

miR134 & miR29a	1.00	85.25	0.00
miR142 & miR29a	1.00	85.25	0.00
miR144 & miR29a	1.00	85.25	0.00
miR150 & miR29a	1.00	85.25	0.00
miR15a & miR29a	1.00	85.25	0.00
miR16 & miR29a	1.00	85.25	0.00
miR181a & miR29a	1.00	85.25	0.00
miR28 & miR29a	1.00	85.25	0.00
miR29a & miR30c	1.00	85.25	0.00
miR29a & miR326	1.00	85.25	0.00
miR29a & miR33a	1.00	85.25	0.00
miR29a & miR486	1.00	85.25	0.00
miR29a & miR744	1.00	85.25	0.00

Supplementary Materials Table S5: Correlations between microRNA levels and immune checkpoint expression in patients with chronic lymphocytic leukemia and secondary immunodeficiency (CLL with SID). Spearman correlation coefficients were calculated for serum concentrations of selected miRNAs (cp/μL) versus percentages of B and T lymphocytes expressing PD-1, PD-L1, CTLA-4, CD200 and CD200R receptors and versus concentrations of their soluble forms (sPD-1, sPD-L1, sCTLA-4, sCD200, sCD200R).

Pair of variables - CLL	R	t(N-2)	p
miR486 & CD8+PD-L1+ [%]	-0.81	-7.39	0.00
miR155 & CD19+PD-L1+ [%]	-0.81	-7.29	0.00
miR486 & CD19+CD200R+ [%]	-0.81	-7.19	0.00
miR155 & CD4+CTLA-4+ [%]	-0.80	-7.13	0.00
miR150 & CD8+CTLA-4+ [%]	-0.80	-7.04	0.00
miR155 & CD4+CD200+ [%]	-0.79	-6.89	0.00
miR486 & CD4+CD200+ [%]	-0.79	-6.72	0.00
miR155 & CD19+CD200R+ [%]	-0.78	-6.63	0.00
miR155 & CD19+CD200+ [%]	-0.78	-6.51	0.00
miR486 & sCD200	-0.78	-6.50	0.00
miR155 & CD19+PD-1+ [%]	-0.77	-6.44	0.00
miR326 & CD8+PD-L1+ [%]	-0.77	-6.43	0.00
miR155 & CD8+PD-1+ [%]	-0.76	-6.27	0.00
miR155 & CD8+CTLA-4+ [%]	-0.76	-6.13	0.00
miR486 & CD8+CD200+ [%]	-0.76	-6.12	0.00
miR155 & sCD200	-0.75	-5.99	0.00
miR486 & CD19+PD-L1+ [%]	-0.75	-5.97	0.00
miR486 & CD4+CTLA-4+ [%]	-0.75	-5.94	0.00
miR155 & CD8+PD-L1+ [%]	-0.74	-5.90	0.00
miR326 & CD4+CD200R+ [%]	-0.74	-5.90	0.00
miR150 & CD8+CD200R+ [%]	-0.74	-5.88	0.00
miR155 & CD4+CD200R+ [%]	-0.74	-5.80	0.00
miR150 & CD4+CTLA-4+ [%]	-0.74	-5.79	0.00
miR326 & CD19+PD-1+ [%]	-0.74	-5.77	0.00
miR326 & sCD200	-0.73	-5.65	0.00
miR326 & sPD-1	-0.73	-5.65	0.00
miR326 & CD19+CTLA-4+ [%]	-0.73	-5.59	0.00
miR486 & sCD200R	-0.72	-5.53	0.00
miR486 & sCTLA-4	-0.72	-5.53	0.00
miR155 & CD8+CD200R+ [%]	-0.72	-5.49	0.00
miR486 & CD19+CTLA-4+[%]	-0.72	-5.44	0.00
miR150 & sPD-L1	-0.72	-5.44	0.00
miR326 & CD4+CD200+ [%]	-0.71	-5.39	0.00
miR150 & CD19+CD200R+ [%]	-0.71	-5.34	0.00
miR155 & CD4+PD-L1+[%]	-0.71	-5.33	0.00
miR486 & CD8+CTLA-4+ [%]	-0.71	-5.32	0.00
miR486 & CD19+PD-1+ [%]	-0.71	-5.30	0.00
miR150 & sCD200R	-0.71	-5.29	0.00
miR155 & CD19+CTLA-4+ [%]	-0.70	-5.26	0.00
miR150 & miR744	-0.70	-5.25	0.00
miR326 & CD19+PD-L1+ [%]	-0.70	-5.24	0.00
miR486 & sPD-1	-0.70	-5.21	0.00
miR486 & CD8+CD200R+ [%]	-0.70	-5.19	0.00
miR150 & CD8+PD-1+ [%]	-0.70	-5.19	0.00
miR155 & sCD200R	-0.70	-5.15	0.00
miR486 & CD4+PD-1+ [%]	-0.70	-5.15	0.00
miR486 & CD4+CD200R+ [%]	-0.70	-5.14	0.00
miR150 & CD8+PDL-1+ [%]	-0.70	-5.13	0.00
miR326 & CD4+PD-L1+ [%]	-0.69	-5.06	0.00
miR326 & CD19+CD200R+ [%]	-0.69	-5.06	0.00
miR150 & CD4+CD200+ [%]	-0.69	-5.02	0.00
miR155 & sPD-1	-0.69	-5.01	0.00
miR326 & CD19+CD200+ [%]	-0.68	-4.96	0.00
miR144 & CD4+CTLA-4+ [%]	-0.68	-4.95	0.00
miR486 & CD19+CD200+ [%]	-0.68	-4.88	0.00
miR326 & CD4+CTLA-4+ [%]	-0.68	-4.87	0.00
miR326 & CD8+CD200R+ [%]	-0.68	-4.87	0.00
miR144 & CD19+CD200+ [%]	-0.67	-4.77	0.00
miR486 & CD8+PD-1+ [%]	-0.66	-4.71	0.00
miR155 & CD4+PD-1+ [%]	-0.66	-4.69	0.00

miR144	& CD19+PD-L1+ [%]	-0.66	-4.67	0.00
miR326	& CD8+CTLA-4+ [%]	-0.66	-4.66	0.00
miR326	& sCD200R	-0.66	-4.64	0.00
miR144	& CD4+CD200+ [%]	-0.65	-4.52	0.00
miR326	& sCTLA-4	-0.65	-4.47	0.00
miR326	& CD8+CD200+ [%]	-0.64	-4.46	0.00
miR155	& sCTLA4	-0.64	-4.42	0.00
miR150	& CD19+PD-L1+ [%]	-0.64	-4.40	0.00
miR486	& CD4+PD-L1+ [%]	-0.64	-4.36	0.00
miR155	& CD8+CD200+ [%]	-0.63	-4.31	0.00
miR144	& CD8+PD-L1+ [%]	-0.63	-4.29	0.00
miR150	& CD19+CD200+ [%]	-0.63	-4.29	0.00
miR150	& sCD200	-0.63	-4.29	0.00
miR150	& CD4+PD-1+ [%]	-0.62	-4.22	0.00
miR150	& sCTLA4	-0.62	-4.16	0.00
miR150	& CD19+PD-1+ [%]	-0.61	-4.10	0.00
miR144	& sCD200	-0.61	-4.05	0.00
miR326	& CD4+PD-1+ [%]	-0.60	-4.02	0.00
miR486	& sPD-L1	-0.60	-4.02	0.00
miR150	& sPD-1	-0.60	-3.97	0.00
miR326	& CD8+PD-1+ [%]	-0.59	-3.90	0.00
miR150	& CD4+PDL-1+ [%]	-0.59	-3.88	0.00
miR144	& sCD200R	-0.59	-3.86	0.00
miR28	& CD19+CTLA-4+ [%]	-0.59	-3.82	0.00
miR181a	& CD19+CTLA-4+ [%]	-0.59	-3.82	0.00
miR144	& CD19+PD-1+ [%]	-0.58	-3.82	0.00
miR150	& CD19+CTLA-4+ [%]	-0.58	-3.79	0.00
miR144	& CD8+CD200+ [%]	-0.58	-3.78	0.00
miR150	& CD4+CD200R+ [%]	-0.58	-3.76	0.00
miR150	& CD8+CD200+ [%]	-0.58	-3.75	0.00
miR144	& CD4+PD-L1+ [%]	-0.58	-3.72	0.00
miR30c	& sCD200R	-0.57	-3.65	0.00
miR144	& CD19+CD200R+ [%]	-0.57	-3.70	0.00
miR144	& CD8+CD200R+ [%]	-0.57	-3.66	0.00
miR30c	& CD19+PD-1+ [%]	-0.57	-3.58	0.00
miR144	& sPD-1	-0.56	-3.59	0.00
miR30c	& CD19+CTLA-4+ [%]	-0.56	-3.52	0.00
miR28	& CD19+CD200R+ [%]	-0.56	-3.56	0.00
miR144	& CD4+CD200R+ [%]	-0.55	-3.48	0.00
miR29a	& sCD200R	-0.55	-3.48	0.00
miR144	& CD4+PD-1+ [%]	-0.54	-3.43	0.00
miR155	& sPD-L1	-0.54	-3.41	0.00
miR181a	& CD19+CD200R+ [%]	-0.54	-3.40	0.00
miR30c	& CD8+CTLA-4+ [%]	-0.54	-3.32	0.00
miR29a	& CD19+CD200+ [%]	-0.54	-3.36	0.00
miR144	& CD8+CTLA-4+ [%]	-0.53	-3.28	0.00
miR28	& CD8+PD-L1+ [%]	-0.52	-3.25	0.00
miR326	& EOS	-0.52	-3.23	0.00
miR150	& miR21	-0.52	-3.22	0.00
miR28	& CD8+CTLA-4+ [%]	-0.52	-3.19	0.00
miR144	& CD8+PD-1+ [%]	-0.51	-3.12	0.00
miR30c	& CD4+CTLA-4+ [%]	-0.50	-3.04	0.01
miR28	& CD19+PD-1+ [%]	-0.50	-3.07	0.00
miR326	& sPD-L1	-0.50	-3.05	0.00
miR144	&sCTLA4	-0.50	-3.04	0.01
miR30c	& sPD-1	-0.50	-2.98	0.01
miR144	& CD19+CTLA-4+ [%]	-0.50	-3.03	0.01
miR28	& CD19+CD200+ [%]	-0.50	-3.03	0.01
miR181a	& CD19+PD-1+ [%]	-0.49	-2.98	0.01
miR181a	& sCD200R	-0.49	-2.96	0.01
miR181a	& sPD-1	-0.49	-2.96	0.01
miR181a	& CD4+CTLA-4+ [%]	-0.48	-2.88	0.01
miR30c	& CD19+CD200+ [%]	-0.48	-2.83	0.01
miR181a	& CD19+CD200+ [%]	-0.48	-2.88	0.01
miR486	& EOS	-0.48	-2.87	0.01
miR30c	& CD19+CD200R+ [%]	-0.47	-2.76	0.01
miR181a	& CD4+PD-L1+ [%]	-0.47	-2.78	0.01
miR155	& miR744	-0.46	-2.77	0.01
miR181a	& CD8+PDL-1+ [%]	-0.46	-2.72	0.01
miR30c	& CD8+PD-L1+ [%]	-0.46	-2.67	0.01
miR181a	& CD4+CD200+ [%]	-0.46	-2.71	0.01
miR181a	& CD19+PD-L1+ [%]	-0.45	-2.69	0.01
miR28	& sPD-1	-0.45	-2.67	0.01
miR28	& CD4+CTLA-4+ [%]	-0.45	-2.65	0.01
miR134	& CD8+PD-L1+ [%]	-0.45	-2.64	0.01
miR28	& CD4+CD3+PD-L1+ [%]	-0.44	-2.61	0.01
miR28	& sCD200R	-0.44	-2.60	0.01
miR30c	& CD8+PD-1+ [%]	-0.44	-2.55	0.02
miR15a	& CD4+CTLA-4+ [%]	-0.43	-2.51	0.02
miR181a	& CD8+PD-1+ [%]	-0.43	-2.49	0.02
miR28	& CD19+PD-L1+ [%]	-0.43	-2.49	0.02
miR21	& miR486	-0.43	-2.49	0.02
miR30c	& CD19+PD-L1+ [%]	-0.43	-2.45	0.02
miR29a	& CD4+CTLA-4+ [%]	-0.42	-2.48	0.02
miR744	& BAS	-0.42	-2.46	0.02
miR30c	& CD4+PDL-1+ [%]	-0.42	-2.41	0.02
miR181a	& sCD200	-0.42	-2.45	0.02
miR28	& CD8+PD-1+ [%]	-0.42	-2.43	0.02
miR134	& CD19+CTLA-4+ [%]	-0.42	-2.42	0.02
miR150	& EOS	-0.41	-2.40	0.02
miR29a	& CD8+CTLA-4+ [%]	-0.41	-2.38	0.02
miR28	& sCD200	-0.41	-2.35	0.03
miR29a	& CD19+CTLA-4+ [%]	-0.41	-2.35	0.03
miR30c	& CD8+CD200+ [%]	-0.40	-2.30	0.03
miR28	& CD4+CD200+ [%]	-0.40	-2.34	0.03
miR29a	& CD19+PD-1+ [%]	-0.40	-2.32	0.03
miR15a	& CD19+CD200+ [%]	-0.40	-2.30	0.03
miR181a	& CD8+CTLA-4+ [%]	-0.40	-2.28	0.03
miR29a	& sCD200	-0.39	-2.27	0.03
miR29a	& CD8+ [%]	-0.39	-2.27	0.03
miR28	& EOS	-0.39	-2.27	0.03
miR30c	& CD4+CD200+ [%]	-0.39	-2.21	0.04

miR125b & miR150	-0.39	-2.25	0.03
miR155 & miR21	-0.39	-2.21	0.04
miR29a & CD8+PD-L1+ [%]	-0.39	-2.21	0.04
miR30c & sCTLA4	-0.38	-2.14	0.04
miR134 & CD19+CD200+ [%]	-0.38	-2.16	0.04
miR15a & CD19+PD-L1+ [%]	-0.38	-2.15	0.04
miR28 & sPD-L1	-0.37	-2.14	0.04
miR15a & CD19+CD200R+ [%]	-0.37	-2.14	0.04
miR134 & EOS	-0.37	-2.12	0.04
miR30c & sPD-L1	-0.37	-2.08	0.05
miR134 & CD19+PD-1+ [%]	-0.37	-2.11	0.04
miR125b & PLT	-0.37	-2.11	0.04
miR221 & BAS	-0.37	-2.10	0.04
miR29a & CD8+PD-1+ [%]	-0.37	-2.10	0.04
miR30c & sCD200	-0.37	-2.06	0.05
miR15a & CD4+CD200+ [%]	-0.37	-2.09	0.05
miR21 & MON	-0.37	-2.07	0.05
miR134 & CD8+CTLA-4+ [%]	-0.36	-2.07	0.05
miR29a & sPD-1	-0.36	-2.07	0.05
miR144 & CD4+ [%]	-0.36	-2.06	0.05
miR144 & EOS	-0.36	-2.05	0.05
miR150 & miR33a	-0.36	-2.05	0.05
miR744 & CD19+CTLA-4+ [%]	0.36	2.05	0.05
miR33a & CD8+PD-1+ [%]	0.37	2.08	0.05
miR326 & BAS	0.37	2.08	0.05
miR142 & miR29a	0.37	2.09	0.05
miR33a & CD8+CD200R+ [%]	0.37	2.12	0.04
miR125b & CD8+CTLA-4+ [%]	0.38	2.14	0.04
miR125b & CD19+PD-L1+ [%]	0.38	2.17	0.04
miR150 & miR28	0.39	2.25	0.03
miR744 & CD19+CD200+ [%]	0.39	2.25	0.03
miR125b & sCD200R	0.39	2.26	0.03
miR142 & miR15a	0.39	2.26	0.03
miR21 & CD19[%]	0.39	2.26	0.03
miR21 & sCD200	0.39	2.27	0.03
miR21 & CD19+PD-1+ [%]	0.40	2.28	0.03
miR744 & CD4+CD200R+ [%]	0.40	2.30	0.03
miR125b & CD4+PD-1+[%]	0.40	2.30	0.03
miR125b & sCD200	0.40	2.31	0.03
miR21 & miR221	0.40	2.31	0.03
miR150 & PLT	0.40	2.32	0.03
miR144 & PLT	0.40	2.33	0.03
miR486 & PLT	0.41	2.41	0.02
miR326 & HGB	0.42	2.42	0.02
miR30c & miR326	0.42	2.39	0.02
miR144 & CD45+ [%]	0.42	2.44	0.02
miR744 & CD4+PD-1+ [%]	0.42	2.45	0.02
miR30c & miR486	0.42	2.41	0.02
miR221 & miR744	0.42	2.47	0.02
miR144 & miR181a	0.42	2.47	0.02
miR744 & sCD200	0.42	2.48	0.02

miR134 & miR142	0.43	2.50	0.02
miR125b & CD8+CD200R+ [%]	0.44	2.57	0.02
miR21 & CD19+CTLA-4+ [%]	0.44	2.57	0.02
miR326 & PLT	0.44	2.57	0.02
miR144 & BAS	0.44	2.58	0.02
miR150 & miR30c	0.44	2.56	0.02
miR134 & miR326	0.44	2.62	0.01
miR155 & miR15a	0.45	2.63	0.01
miR21 & CD4+CD200+ [%]	0.45	2.64	0.01
miR125b & CD8+CD200+ [%]	0.45	2.66	0.01
miR28 & miR326	0.45	2.70	0.01
miR155 & miR30c	0.46	2.66	0.01
miR29a & PLT	0.46	2.73	0.01
miR21 & sCTLA4	0.46	2.76	0.01
miR125b & CD4+CD200R+ [%]	0.46	2.77	0.01
miR33a & sPD-L1	0.46	2.77	0.01
miR744 & CD4+PD-L1+ [%]	0.46	2.77	0.01
miR142 & miR33a	0.47	2.79	0.01
miR181a & miR486	0.47	2.80	0.01
miR125b & sPDL1	0.47	2.82	0.01
miR142 & miR28	0.47	2.82	0.01
miR744 & sPD-1	0.47	2.84	0.01
miR21 & CD8+CTLA-4+ [%]	0.48	2.92	0.01
miR142 & miR181a	0.49	2.94	0.01
miR142 & miR30c	0.49	2.89	0.01
miR21 & CD4+CD200R+ [%]	0.49	2.94	0.01
miR134 & miR33a	0.49	3.00	0.01
miR16 & miR33a	0.49	3.00	0.01
miR744 & CD19+CD200R+ [%]	0.50	3.06	0.00
miR28 & miR486	0.50	3.07	0.00
miR221 & miR33a	0.50	3.07	0.00
miR155 & PLT	0.51	3.11	0.00
miR21 & miR744	0.51	3.12	0.00
miR744 & CD4+CD200+ [%]	0.51	3.12	0.00
miR181a & miR326	0.51	3.13	0.00
miR155 & miR28	0.52	3.20	0.00
miR21 & CD4+CD3+PD-L1+ [%]	0.52	3.24	0.00
miR134 & miR15a	0.52	3.26	0.00
miR744 & CD8+CD200+ [%]	0.53	3.33	0.00
miR21 & CD8+CD200+ [%]	0.54	3.36	0.00
miR125b & miR744	0.54	3.38	0.00
miR744 & CD8+CD200R+ [%]	0.55	3.45	0.00
miR134 & miR29a	0.55	3.46	0.00
miR744 & sCD200R	0.55	3.46	0.00
miR21 & CD4+CD3+PD-1+ [%]	0.55	3.52	0.00
miR21 & sPD-L1	0.56	3.54	0.00
miR21 & CD8+PD-1+ [%]	0.56	3.56	0.00
miR744 & CD8+CTLA-4+ [%]	0.56	3.57	0.00
miR21 & CD19+PD-L1+ [%]	0.56	3.59	0.00
miR134 & miR181a	0.57	3.68	0.00
miR21 & CD19+CD200R+ [%]	0.57	3.69	0.00

miR744 & CD4+CTLA-4+ [%]	0.57	3.71	0.00
miR125b & miR21	0.58	3.76	0.00
miR744 & CD8+PD-1+ [%]	0.58	3.80	0.00
miR125b & CD8+PD-1+ [%]	0.58	3.81	0.00
miR15a & miR29a	0.59	3.88	0.00
miR15a & miR16	0.59	3.88	0.00
miR16 & miR29a	0.60	3.94	0.00
miR150 & miR326	0.60	4.01	0.00
miR155 & miR181a	0.61	4.05	0.00
miR134 & miR30c	0.61	4.02	0.00
miR33a & miR744	0.62	4.21	0.00
miR21 & CD8+CD200R+ [%]	0.63	4.32	0.00
miR125b & miR33a	0.63	4.33	0.00
miR142 & miR16	0.64	4.44	0.00
miR16 & miR142	0.64	4.44	0.00
miR28 & miR29a	0.65	4.48	0.00
miR134 & miR16	0.65	4.55	0.00
miR144 & miR486	0.65	4.55	0.00
miR16 & miR30c	0.66	4.58	0.00
miR21 & miR33a	0.66	4.68	0.00
miR744 & sPDL-1	0.66	4.71	0.00
miR150 & miR486	0.67	4.79	0.00
miR181a & miR29a	0.67	4.82	0.00
miR15a & miR30c	0.70	5.09	0.00
miR15a & miR181a	0.70	5.19	0.00
miR181a & miR30c	0.71	5.19	0.00
miR16 & miR181a	0.72	5.48	0.00
miR134 & miR28	0.73	5.58	0.00
miR16 & miR28	0.75	5.96	0.00
miR29a & miR30c	0.75	5.91	0.00
miR15a & miR28	0.76	6.14	0.00
miR326 & miR486	0.78	6.60	0.00
miR155 & miR486	0.79	6.77	0.00
miR155 & miR326	0.81	7.26	0.00
miR28 & miR30c	0.82	7.44	0.00
miR144 & miR326	0.83	7.85	0.00
miR181a & miR28	0.85	8.65	0.00

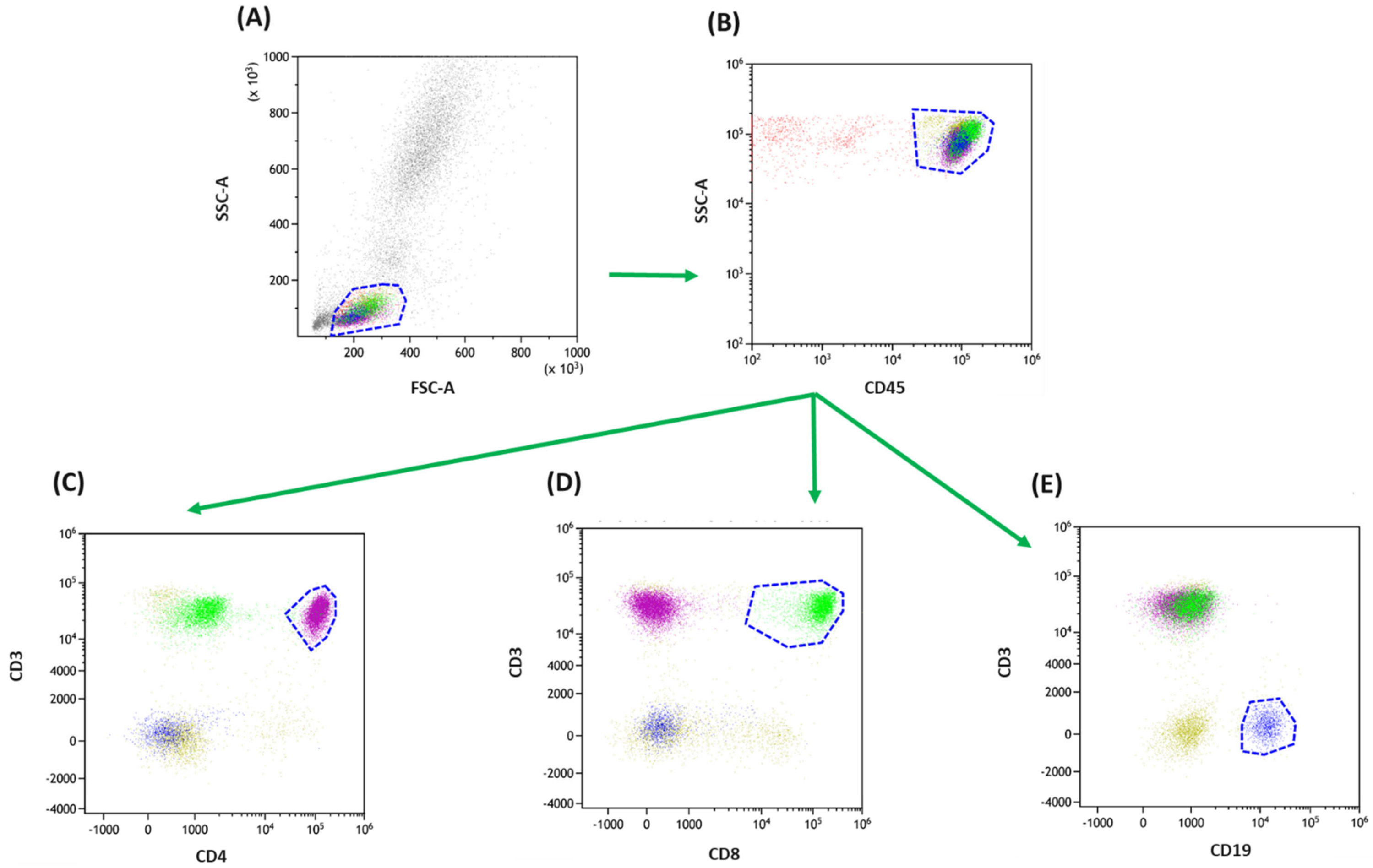

Supplementary Figure 1. The figure illustrates the gating strategy used to analyze immune cell subpopulations by flow cytometry. In the first step (A), the lymphocyte population was selected based on light scatter properties – FSC-A (a measure of cell size) and SSC-A (a measure of granularity). In the next step (B), these lymphocytes were further assessed for the expression of CD45, a marker typical of leukocytes. In the SSC-A vs. CD45 plot, the gate identifies CD45+ cells, representing the white blood cell population. Panel (C) presents the analysis of T helper lymphocytes (CD3+CD4+ cells). In the CD4 vs. CD3 plot, the double-positive population is marked in violet. Panel (D) focuses on cytotoxic T lymphocytes (CD3+CD8+ cells), with the corresponding double-positive population marked in green in the CD8 vs. CD3 plot. Panel (E) shows the identification of B lymphocytes (CD19+CD3− cells), which are negative for CD3 but positive for the B-cell marker CD19 and are gated in blue.

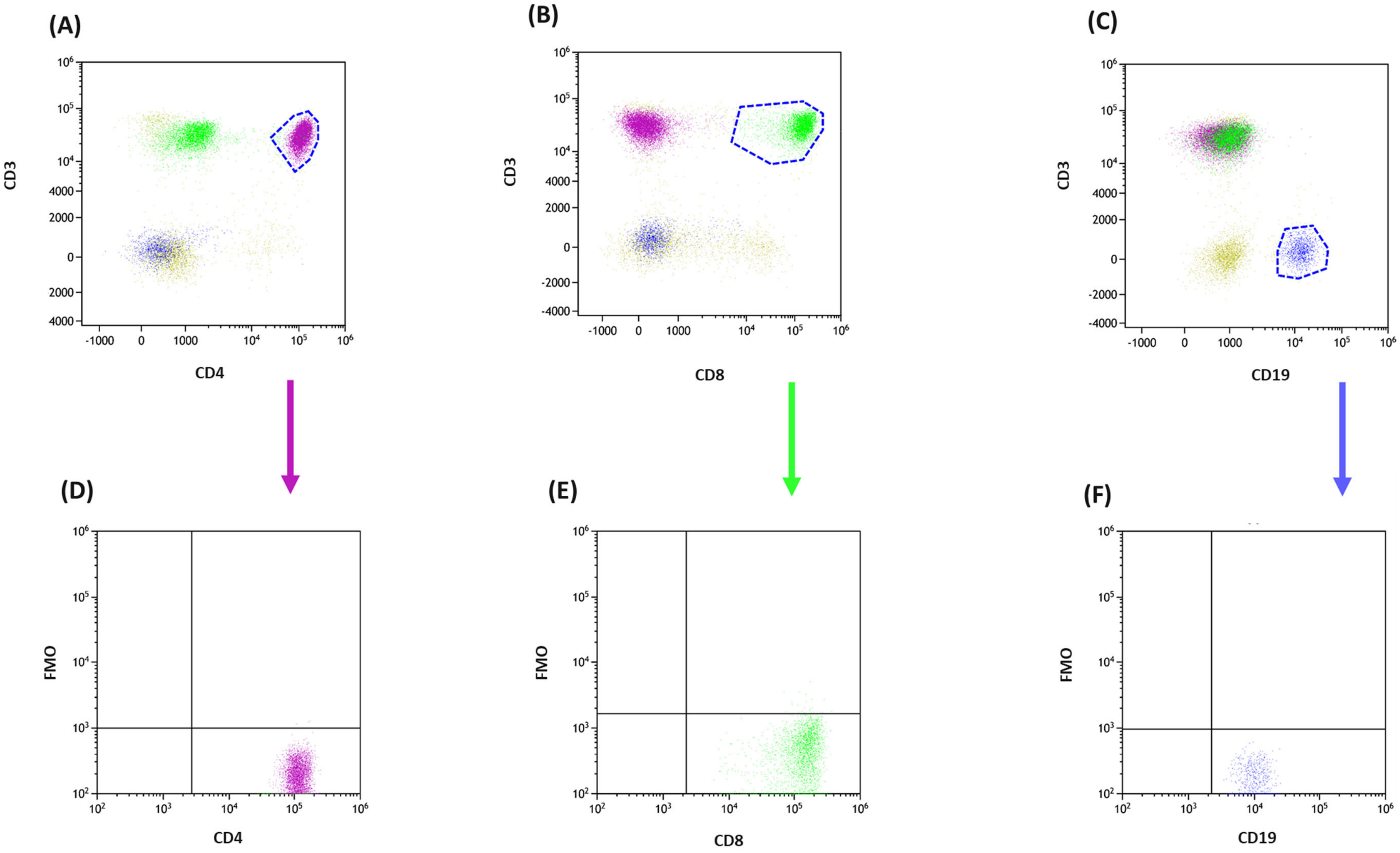

Supplementary Figure 2. The figure shows selected lymphocyte subpopulations studied by flow cytometry and the setting of cutoff points using FMO controls. (A–C) Gating strategy for identifying $CD3^+CD4^+$ T cells (A), $CD3^+CD8^+$ T cells (B), and $CD3^-CD19^+$ B cells (C). Fluorescence Minus One (FMO) controls for CD4 (D), CD8 (E), and CD19 (F) allow for setting the cutoff point for the studied markers.
